# Precision Oncology in Non-small Cell Lung Cancer: A Comparative Study of Contextualized ChatGPT Models

**DOI:** 10.7759/cureus.81097

**Published:** 2025-03-24

**Authors:** Ethan D L Brown, Harshal A Shah, Brianna M Donnelly, Max Ward, Morana Vojnic, Randy S D'Amico

**Affiliations:** 1 Neurological Surgery, Donald and Barbara Zucker School of Medicine at Hofstra/Northwell, Hempstead, USA; 2 Oncology, Donald and Barbara Zucker School of Medicine at Hofstra/Northwell, Hempstead, USA

**Keywords:** driver alterations, large language model, nsclc, precision oncology, targeted therapy

## Abstract

Objectives: The growing adoption of Large Language Models (LLMs) in medicine has raised important questions about their potential utility for clinical decision support within oncology. This study aimed to evaluate the effects of various contextualization methods on ChatGPT’s ability to provide National Comprehensive Cancer Network (NCCN) guideline-aligned recommendations on managing non-small cell lung cancer (NSCLC).

Methodology: GPT-4o, base GPT-4, and GPT-4 models contextualized with prompts and PDF documents were asked to identify preferred chemotherapies for twelve advanced lung cancers given molecular profiles derived from the 2024 NCCN Clinical Practice Guidelines in Oncology for NSCLC. GPT responses were subsequently compared to NCCN guidelines using readability scores and qualitative reviewer assessments of (1) recommendation of specific targeted therapy, (2) agreement with NCCN-guideline-preferred therapies, (3) recommendation of guideline non-concordant therapies, and (4) provision of supplementary information.

Results: The PDF+Prompt contextualized model demonstrated elevated agreement scores of 23/24 versus 17/24 for GPT-4 (*P *= 0.040) and 18/24 for GPT-4o (*P *= 0.089). No PDF+Prompt model responses contained guideline non-concordant therapies in contrast to 4/12 responses for GPT4 (*P *= 0.093) and 5/12 responses for GPT4o (*P *= 0.037). Comparison of response readability between the PDF+Prompt model and GPT-4 or GPT-4o showed a lower mean word count (both *P* < 0.001), Simple Measure of Gobbledygook (SMOG) score (both *P* < 0.001), and Gunning Fog readability score (*P* < 0.001 for GPT-4, *P* = 0.002 for GPT-4o). Prompting alone did not significantly improve agreement or reduce the rate of non-concordant therapy recommendations.

Conclusions: The performance gains observed following contextualization suggest that broader applications of LLMs in oncology may exist than current literature indicates. This study provides proof of concept for the use of contextualized GPT models in oncology and showcases their accessibility. Future studies validating this application within additional cancer types or real-life patient encounters could provide an important bridge to eventual adoption.

## Introduction

Non-small cell lung cancer (NSCLC) remains a significant global health challenge. Over the past two decades, oncology has experienced a rapid shift toward precision medicine, where small molecule therapies are tailored to target specific genetic mutations in patient-specific cancers. Targeted therapeutics have been critical for improving outcomes in NSCLC. However, this evolution in treatment paradigms has necessitated a high level of expertise in selecting the appropriate therapeutic agents, given the ever-expanding array of possible mutations and corresponding treatment options [1].

As artificial intelligence (AI) continues to advance, large language models (LLMs) like OpenAI's ChatGPT have demonstrated surprising capabilities in navigating technical subject matter. These models, trained on vast datasets composed of both publicly and privately available information, have quickly gained attention in the medical community. Their potential applications span across diagnosis, treatment, and ethical considerations, prompting a surge in research investigating their utility within various medical fields, including oncology due to their obvious implications for precision medicine [2-4]. Despite the promising potential of LLMs, their performance in making complex medical diagnoses and treatment recommendations has been met with mixed results [5,6]. Studies have shown that while ChatGPT can achieve remarkable accuracy in answering certain medical questions, its efficacy diminishes when confronted with more challenging or specialized queries. These limitations are especially evident in the context of personalized oncology, where the heterogeneity of molecular profiles presents a formidable challenge [5].

In oncology, the implications of these limitations are profound. Studies comparing GPT-3.5's ability to recommend treatments for molecular profiles outside of established guidelines have revealed considerable discrepancies between AI-generated and oncologist-recommended therapies. These findings underscore the complexity of personalized cancer care, where clinical practice often demands the narrowing of several plausible therapeutic options to a single preferred therapy. The tendency of LLMs to generate an excessive number of treatment recommendations further complicates their clinical utility, as it may lead to non-concordant and potentially inappropriate therapeutic choices [7-10].

One possible remedy may lie in the contextualization of generalized LLMs around domain-specific knowledge using targeted prompting or reference documents. Previous studies in neurosurgery, otolaryngology, and radiology have shown that integrating clinical guidelines, expert consensus statements, or relevant prompts can improve the ability of these devices to provide clinically accurate recommendations [10-13]. This approach is now easily replicable, as OpenAI allows the user to create GPT-4-based contextualized models within the ChatGPT user interface. Moreover, contextualization may be particularly well suited to preventing excessive ChatGPT recommendations given that it may improve the conciseness of GPT responses [13].

This study seeks to explore the potential of contextualized GPT models in the management of advanced or metastatic NSCLC. In other words- can contextualization improve ChatGPT's accuracy in NSCLC therapy recommendation? Specifically, we compare the performance of three configurations: the standard GPT-4 model, the latest release GPT-4o variant, and a contextually enhanced GPT-4 model architecture that integrated PDF-based documents and tailored prompts. We hypothesized that contextualized GPT models would yield more accurate and guideline-concordant therapy recommendations, potentially improving the precision of AI-assisted decision-making in complex oncological cases.

## Materials and methods

**Figure 1:**
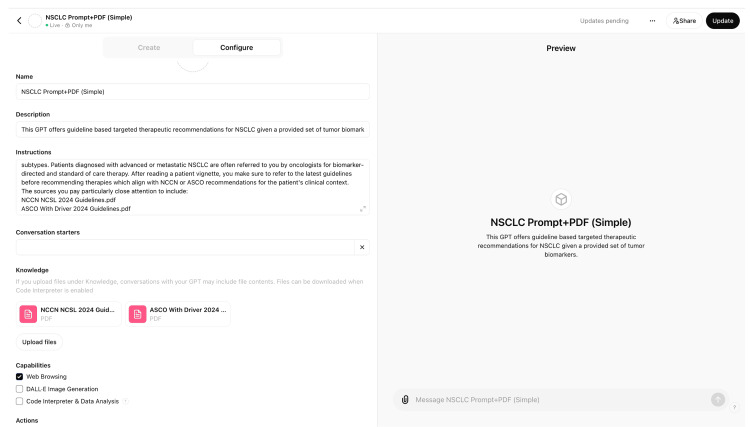
Creating contextualized GPTs. The Build a GPT configuration page for the GPT+PDF+Prompt model is shown with injected PDF documents as well as written instructions.

Two contextualized GPTs were created using the *Create a GPT* functionality of ChatGPT Plus. These models were configured with specific *Instructions* and/or *Knowledge* as outlined below and were equipped with web browsing capabilities. The models were designed to be trained either on written instructions alone (Prompt GPT-4) or on a combination of PDF materials and written instructions (PDF+Prompt GPT-4), as illustrated in Figure 1.

PDF materials were carefully selected following a review of guidelines from the Congress of Neurological Surgeons (CNS), the National Comprehensive Cancer Network (NCCN), the Society for Neuro-Oncology (SNO), the American Society of Clinical Oncology (ASCO), and the European Society for Medical Oncology (ESMO) published within the past five years, specifically addressing the use of targeted therapeutics for advanced or metastatic NSCLC. The selected materials included (1) *Therapy for Stage IV Non-Small Cell Lung Cancer with Driver Alterations: ASCO Living Guideline, Version 2023.3*, and (2) *Non-Small Cell Lung Cancer, Version 5.2024, NCCN Clinical Practice Guidelines in Oncology* (Sections NSCL 19-36, NSCL-H, NSCL-I, NSCL-J, NSCL-K, CAT-1, MS 7-24, and 74-97) [14,15]. This targeted approach to training materials was used to closely align contextualized and assessed topics, as it has been previously suggested that training on unrelated materials can predispose to *hallucinations* [13]. Model weights and configurations were not adjusted from the indicated settings on OpenAI's portal.

The written instructions provided to the models included directives such as “make sure to refer to the latest guidelines”, “[recommend] therapies which align with the NCCN or ASCO recommendations for this patient’s clinical context”, “[provide] concise therapy recommendations”, “emphasize preferred therapies”, and “do not include redundant or inferior treatments.” A complete copy of training instructions can be identified in Appendix A.

To create test questions for the ChatGPT models, biomarker-directed therapy recommendations for advanced or metastatic NSCLC were retrieved from the NCCN Guidelines, Version 6.2024 (Appendix B). Modified queries were derived from the preferred therapies listed in Sections NSCL 21-34 and from Category 1 recommendations when no preferred therapy existed (Appendix C). These queries assessed preferred therapies for various driver alterations, including epidermal growth factor receptor (EGFR) mutations, Kirsten Rat Sarcoma Viral Oncogene Homolog (KRAS) mutations, anaplastic lymphoma kinase (ALK) rearrangements, ROS Proto-Oncogene 1 (ROS1) rearrangements, B-Raf Proto-Oncogene (BRAF) mutations, Neurotrophic Tyrosine Receptor Kinase (NTRK) gene fusions, METex14 skipping mutations, REarranged during Transfection (RET) gene rearrangements, and ERBB2 mutations. These queries were then input into GPT-4, GPT-4o, Prompt GPT-4, and PDF+Prompt GPT-4 models. The standard GPT-4 model was included to provide a baseline comparison, as at the time of this study, the contextualized models were built on the GPT-4 architecture rather than the newer GPT-4o. To maximize reproducibility, new ChatGPT sessions were initiated for each query. Responses were recorded verbatim, and the session was closed before initiating a new query. All responses were collected on June 30, 2024. A complete record of model responses is presented within the supplemental materials (Appendices D-G). 

ChatGPT responses were assessed qualitatively by two human reviewers (BD, HS) for coherence with the source recommendations. Responses were graded as either *yes* or *no* for (1) providing a specific targeted therapy recommendation, (2) suggesting therapies that were unmentioned by NCCN for a given context, and (3) providing supplemental information to support its therapy recommendation [16]. Responses were subsequently graded for alignment with NCCN therapy recommendations “no (0)”, “partial (1)”, or “complete (2)” alignment [13,17]. A score of 0 was assigned if no guideline-concordant therapies were suggested, 1 if any GPT-preferred therapy recommendation was guideline-concordant, and 2 if GPT-preferred therapy recommendations were both guideline-concordant and NCCN “preferred.” NCCN defines preferred therapies as those “based on superior efficacy, safety, and evidence and, when appropriate, affordability” [18]. For recommendations without an NCCN-designated “preferred therapy,” a score of 2 could still be attained if all GPT-preferred therapies were present in the guidelines.

Reviewer discrepancies were resolved by a third reviewer (EB). The level of agreement was compared across models using the Mann-Whitney U test with a significance level of 0.05. Other qualitative metrics were compared using Fisher's Exact test with a significance level of 0.05. Cohen's kappa (κ) was subsequently calculated to measure the inter-rater reliability of primary reviewers for concordance and non-NCCN therapy recommendations. Readability between outputs was compared using validated tools, including the Flesch-Kincaid Reading Ease Score, Gunning Fog Index, Simple Measure of Gobbledygook (SMOG), and word count [19]. These readability metrics were selected to ensure the comparability of our responses to those of previously published investigations of oncology and quantify any direct effects of contextualization on response metrics [19-21]. All readability metrics were calculated in R (version 4.3.2) using the “quanteda” and “quanteda.textstats” packages and compared across models using two-tailed t-tests with an alpha level of 0.05. Quantitative data are displayed as mean and standard deviation (SD).

An Institutional Review Board review was not required as the study did not involve human participants. To maximize reproducibility, this study complied with the Strengthening Reporting of Observational Studies in Epidemiology (STROBE) Checklist for observational studies wherever possible.

## Results

Twelve treatment recommendations from the NCCN Guidelines Version 6.2024 for NSCLC were modified into questions for this study. Each model returned responses for all modified queries. The introduction of context, whether through prompts or PDFs and prompts, produced contextualized model responses, which were syntactically and semantically distinct from those of GPT-4 (Table 1).

**Table 1:** ChatGPT-4 and contextualized GPT response comparisons. Several versions of GPT-4 were prompted with the modified prompt *Given an advanced lung adenocarcinoma patient with no prior treatment and CD74-ROS1 fusion mutations. What therapy or therapies would be preferred?*, yielding evident differences in response output. First-line therapy referenced by NSCL-30 NCCN Clinical Practice Guidelines in Oncology (NCCN Guidelines) for Non-Small Cell Lung Cancer, Version 6.2024 for treating ROS1 rearrangement is included for comparison. A complete collection of prompts and model responses can be found in Appendices C-G. TKI, tyrosine kinase inhibitor; NSCLC, non-small cell lung cancer; NCCN, National Comprehensive Cancer Network; ROS1, ROS Proto-Oncogene 1; ALK, anaplastic lymphoma kinase; NTRK, Neurotrophic Tyrosine Receptor Kinase

Model	Response
Modified Prompt	Given an advanced lung adenocarcinoma patient with no prior treatment and CD74-ROS1 fusion mutations. What therapy or therapies would be preferred?
Consensus Statement	For ROS1 rearrangement discovered before first-line systemic therapy: Preferred: Entrectinib or Crizotinib or Repotrectinib or Other Recommended: Ceritinib
GPT-4	For an advanced lung adenocarcinoma patient with the CD74-ROS1 fusion mutation who has not previously been treated, the preferred therapeutic approach typically centers on targeted therapy using ROS1 inhibitors. ROS1 is a receptor tyrosine kinase, and mutations involving ROS1 fusions can drive cancer development by activating signaling pathways that promote cell growth and survival. Here are some key points and treatment options: Crizotinib: This is an oral small-molecule inhibitor that targets ROS1 and is effective in treating ROS1-positive non-small cell lung cancer (NSCLC). It was the first ROS1 inhibitor approved by the FDA for this purpose based on the results of clinical trials demonstrating significant tumor response rates and progression-free survival benefits. Other ROS1 Inhibitors: Additional targeted therapies for ROS1-positive NSCLC include: Entrectinib: This drug is a newer generation inhibitor that also targets NTRK and ALK in addition to ROS1. It is particularly noted for its ability to penetrate the central nervous system, making it useful in cases where there is brain involvement. Lorlatinib: Another option for patients, particularly those who may have developed resistance to earlier treatments like Crizotinib. Lorlatinib is a third-generation inhibitor that targets ALK and ROS1 and has shown activity against brain metastases. Monitoring and Management of Resistance: While ROS1 inhibitors can be highly effective, resistance to therapy often develops. It is important to monitor the patient’s response and consider biopsies or liquid biopsies to detect resistance mutations. This can guide the choice of subsequent lines of therapy, including switching to different ROS1 inhibitors or combining them with other treatments. Clinical Trials: Given the rapidly evolving landscape of targeted therapies, participation in clinical trials can be considered to access novel therapeutic options that might be beneficial. Comprehensive Care: Beyond targeted therapies, the management of an advanced lung cancer patient should include supportive care to manage symptoms, improve quality of life, and address any complications of the disease or its treatment. Treatment must be guided by an oncologist specializing in lung cancer, who can tailor therapy choices to the specific genetic profile and overall health status of the patient. Regular follow-ups and adjustments in the treatment plan based on the response to therapy and evolving clinical evidence are key components of effective management.
GPT-4o	For a patient with advanced lung adenocarcinoma harboring a CD74-ROS1 fusion mutation and no prior treatment, the preferred therapies are typically targeted therapies that specifically inhibit ROS1. The following therapies are recommended based on current clinical guidelines and evidence: Crizotinib: This is often the first-line treatment for patients with ROS1-positive lung adenocarcinoma. Crizotinib is a TKI that targets ALK, ROS1, and MET. It has shown significant efficacy in patients with ROS1 rearrangements. Entrectinib: Another targeted therapy option, Entrectinib is also a TKI that inhibits ROS1, TRK, and ALK. It is approved for the treatment of ROS1-positive non-small cell lung cancer (NSCLC) and has shown efficacy in both systemic disease and brain metastases. Lorlatinib: This TKI is primarily known for its use in ALK-positive NSCLC, but it is also effective against ROS1-positive lung cancer. Lorlatinib can be considered especially in cases where the cancer has progressed on Crizotinib, due to its ability to penetrate the blood-brain barrier and its activity against certain ROS1 resistance mutations. In addition to these targeted therapies, comprehensive genomic profiling should be considered to identify any other potential mutations or alterations that could influence treatment decisions. Close monitoring for response to therapy and any emerging resistance mutations is also crucial, as it may necessitate a change in therapeutic strategy. These therapies are preferred due to their targeted mechanism of action, which tends to result in higher response rates and more favorable side effect profiles compared to traditional chemotherapy.
GPT-4+ Prompt	For an advanced lung adenocarcinoma patient with no prior treatment and CD74-ROS1 fusion mutations, the preferred therapies are: Entrectinib Crizotinib; evidence supporting the recommendation: Entrectinib is an ROS1 inhibitor with demonstrated efficacy in ROS1-positive NSCLC, showing high response rates and durability of response. Citation: NCCN Guidelines, Version 6.2024 Crizotinib Evidence Supporting the Recommendation: Crizotinib, another ROS1 inhibitor, is effective in ROS1-rearranged NSCLC, with substantial clinical benefit and prolonged progression-free survival. Citation: NCCN Guidelines, Version 6.2024 These recommendations are based on the latest NCCN guidelines, which highlight both Entrectinib and Crizotinib as preferred options for patients with ROS1 rearrangements.
GPT-4+ PDF+ Training	For a patient with advanced lung adenocarcinoma with a CD74-ROS1 fusion mutation and no prior treatment, the preferred therapies according to the latest guidelines are: Crizotinib Evidence Supporting the Recommendation: Crizotinib has demonstrated significant efficacy in ROS1-positive NSCLC patients. Multiple studies have shown an objective response rate (ORR) of approximately 70%-80%, with median progression-free survival (PFS) ranging from 15.9 to 20 months and median overall survival (OS) of 51.4 months. Citation: NCCN NCSL 2024 Guidelines​​. Entrectinib Evidence Supporting the Recommendation: Entrectinib has shown a high ORR of 77% in ROS1-positive NSCLC patients, with a median PFS of 15.7 months. It is particularly noted for its effectiveness in patients with CNS metastases. Citation: NCCN NCSL 2024 Guidelines​​. Repotrectinib Evidence Supporting the Recommendation: Repotrectinib is a next-generation ROS1 inhibitor that has shown a confirmed ORR of 79% in ROS1 TKI-naïve patients and 38% in those previously treated with a ROS1 TKI. The median PFS was 35.7 months in TKI-naïve patients. Citation: NCCN NCSL 2024 Guidelines​​. These therapies are recommended based on their efficacy and the clinical trial data supporting their use in ROS1-positive NSCLC patients.

The responses from each model were subsequently assessed by reviewers (Table 2). Among the models, only GPT-4o failed to provide a specific targeted therapy for any of the queries. The standard GPT-4 model produced responses with 58.3% partial agreement and 41.7% complete agreement with NCCN guidelines. GPT-4o responses showed 50% partial agreement and 50% complete agreement. In contrast, the Prompt GPT-4 model responses showed 8.4% partial agreement and 91.6% complete agreement while the PDF+Prompt GPT-4 model had 16.8% partial agreement and 83.2% complete agreement. A Mann-Whitney U test revealed that the PDF+Prompt model had significantly higher agreement with NCCN guidelines compared to GPT-4 (*P* = 0.040), although this increase was not statistically significant when compared to GPT-4o (*P* = 0.089). However, the PDF+Prompt model was significantly less likely to recommend guideline-non-concordant therapies than GPT-4o (0 vs. 5 non-concordant therapies, *P *= 0.0373). Additionally, all models universally provided supplementary information related to the identified therapies. Cohen's κ for inter-rater reliability between primary reviewers was 0.688 for non-NCCN therapy recommendations, indicating substantial agreement, and 0.417 for model concordance, indicating moderate agreement.

**Table 2:** Qualitative comparison of GPT responses. Consensus reviewer gradings of the twelve GPT-4 responses in domains of prompt answering, congruence with NCCN preferred therapies, inclusion of non-NCCN referenced therapies, and presence of relevant supplementary info are shown. While prompt answering and supplementary information were assessed as 0 or 1, agreement with expert consensus was graded using a 0-2 scale. Statistical tests comparing trained to untrained models are shown with values derived from the Mann-Whitney U test for expert agreement and the Fisher’s Exact test for non-NCCN recommendations. NCCN, National Comprehensive Cancer Network

Model	Specific targeted therapy recommendation	Agreement with expert consensus (0-2)	Versus GPT-4 test statistic (*P*-value)	Versus GPT-4o test statistic (*P*-value)	Non-NCCN therapy	Versus GPT-4 *P*-value	Versus GPT-4o *P*-value	Relevant supplementary info
GPT-4	12/12	17/24	-	-	4/12	-	-	12/12
GPT-4o	11/12	18/24	-	-	5/12	-	-	12/12
GPT-4+Prompt	12/12	22/24	42 (.089)	48 (.174)	2/12	.640	.371	12/12
GPT-4+PDF+Prompt	12/12	23/24	37 (.040)	42 (.089)	0/12	.093	.037	12/12

**Figure 2:**
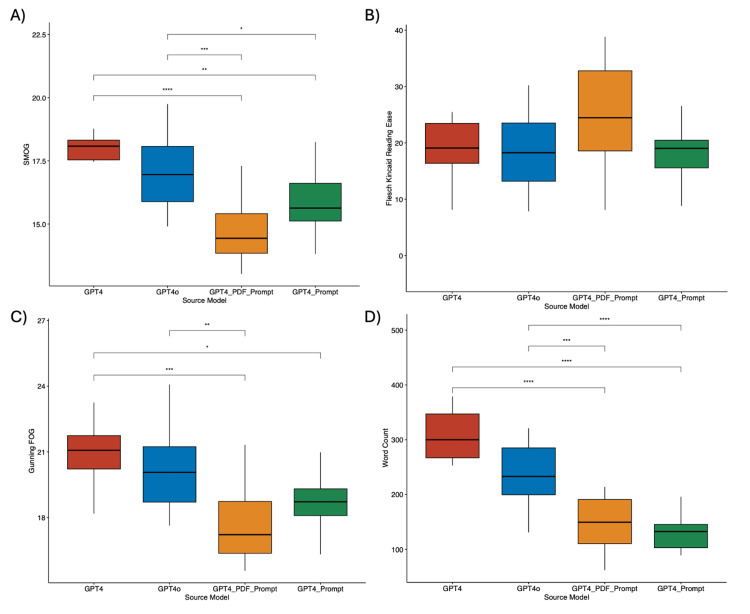
Readability comparison between various GPT-4 models. Box and whisker plots comparing readability metrics comparing base and contextualized models are shown. Readability metrics compared include (A) Simple Measure of Gobbledygook (SMOG), (B) Flesch Kincaid Reading Ease, (C) Gunning FOG, and (D) Word Count. Single, double, triple, and quadruple asterisks denote a difference in readability metrics of *P *< 0.05, *P* < 0.01, *P* < 0.001, and *P* < 0.0001, respectively, using two-tailed t-tests.

To assess the effects of contextualization on the readability of GPT outputs, various measures were calculated, including the Flesch Kincaid Reading Ease Score, Gunning Fog Index, SMOG, and word count (Figure 2). Contextualized model responses demonstrated significantly reduced average word count relative to native GPT-4 (mean 308, SD 46.6) and GPT-4o (mean 237, SD 54.8). Specifically, the PDF+Prompt model had a mean (SD) of 148 (53.0) words per response (*P* < 0.001), while the Prompt model averaged 130 (32.0) words per response (*P* < 0.001). Flesch Reading Ease scores did not differ significantly between models, with mean (SD) values of 19.8 (7.5), 17.2 (9.4), 24.3 (10.6), and 17.9 (5.1) for the GPT-4, GPT-4o, Prompt+PDF, and Prompt models, respectively. Mean SMOG scores were 17.7 (1.3), 17.1 (1.6), 14.7 (1.3), and 15.9 (1.4) for the GPT-4, GPT-4o, Prompt+PDF, and Prompt models, respectively. Meanwhile, mean Gunning Fog scores were 20.6 (1.8), 20.2 (2.0), 17.6 (1.7), and 18.8 (1.6) for GPT-4, GPT-4o, Prompt+PDF, and Prompt models, respectively. SMOG and Gunning Fog reading levels were lower in PDF+Prompt compared to both GPT-4 (all *P *< 0.001) and GPT-4o (*P* < 0.001, *P *= 0.002). Similarly, the Prompt model demonstrated lower SMOG (*P *= 0.003) and Gunning Fog (*P *= 0.014) scores than GPT-4, as well as lower SMOG scores than GPT-4o (*P *= 0.049).

## Discussion

This study is the first to evaluate the impact of contextualization on the efficacy of ChatGPT’s therapeutic recommendations using OpenAI’s Build a GPT tool in the context of NSCLC. Specifically, we focused on advanced or metastatic NSCLC and assessed how contextualization with domain-specific PDFs and tailored prompts influenced the concordance of AI-generated recommendations with established NCCN guidelines. Our findings indicate that contextualized GPT models - especially those combining PDFs and prompts - tended to improve guideline adherence and reduce the rate of non-concordant therapy recommendations compared to the standard GPT-4 and GPT-4o models. Additionally, contextualization resulted in notable changes in the structure and readability of the responses, including significant decreases in word count, SMOG, and Gunning Fog readability scores. 

Impact of contextualization on GPT responses

The introduction of context, whether through prompts alone or in combination with PDFs, appeared to significantly refine the quality of the GPT model's outputs. Contextualized models generally produced more concise and focused therapy recommendations, as evidenced by the reduced word count. For instance, in response to a query about a lung adenocarcinoma patient with ERBB2 mutations showing recurrence after treatment with cisplatin and pemetrexed, the standard GPT-4 model suggested multiple therapies, including trastuzumab, afatinib, docetaxel, and pembrolizumab, alongside additional commentary on clinical trials, further genomic profiling, and palliative care. In contrast, the PDF+Prompt model succinctly recommended trastuzumab deruxtecan as the preferred therapy and provided supporting evidence for its choice.

These findings suggest that contextualization not only enhances the relevance of therapy recommendations but also helps streamline the information, making it more actionable for clinicians. Interestingly, while base GPT models did not include citations, contextualized models attempted to do so, although the citation formats were inconsistent. This attempt at citation integration aligns with previous observations that GPT models, when provided with domain-specific information, can better mimic expert behavior, albeit with some limitations. The significantly lower Gunning Fog and SMOG scores observed from contextualized models may be an artifact of this process as previous studies have noted no differences in readability between base GPT and contextualized models [13].

Guideline concordance and therapy recommendations

A key observation from our study was that the base GPT models frequently suggested therapies not recommended by the NCCN, whereas the contextualized models were more likely to incorrectly omit certain guideline-concordant therapies. For example, the Prompt, GPT-4, and GPT-4o models all recommended mobocertinib, an EGFR tyrosine kinase inhibitor, for the treatment of EGFR exon 20 insertions, despite its withdrawal from the U.S. market in 2023 and explicit non-recommendation by the NCCN. Conversely, in its sole error in the assessed queries, the PDF+Prompt model recommended only Amivantamab for treatment of an EGFR exon 20 insertion adenocarcinoma, while neglecting to include carboplatin or pemetrexed.

This pattern suggests that while contextualization can reduce the over-recommendation of therapies-a common issue with LLMs-it might also lead to the under-recommendation of viable options. The degree to which this reduction in therapy recommendations can be modulated using different contextual inputs remains to be explored. However, this behavior could serve as a useful counterbalance to the well-documented tendency of LLMs to over-recommend treatments, which has been highlighted in multiple studies [7-9]. The adaptability of LLMs to different strategies for contextualization is an important area for future study, as no technical barriers prevent institution- or clinician-specific protocols from being used in place of clinical guidelines. The dynamic nature of oncology practice is another consideration, as AI models will need to be continually refined to remain current.

Ethical considerations will be an important consideration for any real-world implementation of clinical AI models, as concerns like privacy, evolving training materials, appropriate patient access, and proper clinician use remain to be solved in the coming years [3]. The rapidly evolving technological landscape could present challenges for any such analysis, as the varying performance of contextualized and non-contextualized LLMs across medical specialties and scenarios will require granular consideration [13,22]. However, given its ease of use and increasing ubiquity, clinicians must take the lead in driving the medical applications of LLMs rather than merely reacting to their inconsistent adoption by concerned patients.

Comparative analysis with existing literature

The significant heterogeneity of molecular profiles in personalized oncology has left previous research exploring the possible efficacy of LLMs with mixed results. In a study examining the ability of LLMs to recommend treatments for molecular profiles outside of guideline recommendations, Benary et al. found that GPT-3.5 had an F1 score of only 0.17 compared to an oncologist comparator [7]. In a similar study comparing GPT-3.5 recommendations to those of NCCN guidelines, Chen et al. found that while all responses contained at least one guideline-concordant recommendation, 34.3% contained one or more non-concordant recommendations as well [8]. Some of these shortcomings may be due to an excessive number of treatment recommendations, as Schulte found that GPT-3.5 suggested more therapies than NCCN for 70% of guideline-based vignettes, leaving 23% of ChatGPT therapies non-concordant [9]. Benary et al.’s study has similar findings, with a median of 7.5 treatments per patient for GPT-3.5 versus 4 for the reference standard [7]. Thus, even though many plausible therapeutic options must be narrowed to a preferred regimen in actual clinical practice, in consultation with a molecular tumor board or otherwise, base model LLMs have consistently proffered scattershot recommendations [10]. The limited investigations of contextualized models have identified possible improvements, with Ferber et al. finding improved GPT-4 concordance (84% contextualized vs. 57% base model) with ESMO guidelines on pancreatic, hepatocellular, and colorectal cancer following retrieval-augmented generation with relevant guidelines [23]. Our study extends these findings to NSCLC and identifies novel effects of contextualization on response readability.

Moreover, our findings corroborate previous studies by documenting a roughly 30% rate of non-concordant therapy recommendations in the base GPT model responses [8,9]. Similarly, we observed limited instances of cited evidence within GPT-4 and GPT-4o responses, with contextualized models providing non-specific citations [7]. Interestingly, some novel recommendations by GPT, although not mentioned in NCCN guidelines, were clinically plausible. For example, the Prompt model suggested a combination of Amivantamab and Lazertinib for treating EGFR L858R mutations. Although not included in our initial reference guidelines, this regimen has recently been rated as a high-evidence intervention by the latest ASCO Living Guidelines, demonstrating the potential utility of contextualized GPTs in rapidly evolving fields like precision oncology [24].

Importantly, this study demonstrates that despite the potential accuracy improvements associated with specialized LLM models, clinicians should continue to interpret AI-generated recommendations with caution due to their narrower recommendation profile. To ensure safe deployment any clinical models should overseen by a clinician informed about external factors that may affect therapy preference such as the availability of clinical trials and patient preferences. While the gold standard criterion should always be that of a molecular tumor board, these data suggest that contextualized GPTs could yet serve a useful role in the tailoring of precision oncology treatment regimens. 

Limitations

Several limitations of our study should be acknowledged. First, our queries were derived exclusively from guidelines for advanced NSCLC and focused on therapies with substantial supporting evidence. This limited scope may have left our study underpowered to detect subtle differences between the models and may also restrict the generalizability of our findings to other oncologic contexts such as colorectal or hematologic malignancies. Additionally, our vignettes provided only the molecular profile and limited clinical history, which does not fully replicate the complexity of real-world clinical scenarios. In practice, the integration of AI tools into clinical workflows would likely involve more comprehensive data inputs, including electronic health records and broader clinical context clinical context [25]. Thus, additional contextualization, along with the integration of U.S. Food and Drug Administration (FDA)-recognized precision oncology databases, which were not included in this study due to platform restrictions on AI model training, could enhance the accuracy of AI-generated recommendations in future applications. However, it must be noted that as LLM outputs are inherently random, the effects of contextualization require further study to conclusively determine their effect on guideline concordance. Finally, inter-rater reliability according to Cohen's κ was only moderate for model concordance, indicating possible challenges inherent to grading free model responses according to pre-specified written guidelines.

Future studies could expand on our work by exploring a wider range of pathologies and employing reference criteria that better reflect the uncertainties of clinical practice, such as the deliberations of molecular tumor boards. Additionally, investigating the integration of FDA-recognized biomarker databases could provide valuable insights into how AI models might be optimized for clinical use. Similarly, analysis of model performance by cancer subtype within larger cohorts could help delineate possible causes of bias within model responses. Given the rapid evolution of LLMs and the proprietary nature of tools like ChatGPT, ongoing validation studies will be essential to ensure the continued relevance of our findings.

## Conclusions

Our study found that contextualized ChatGPT models may experience increased alignment with NCCN-preferred therapies, reduced rate of non-guideline concordant recommendations, and alterations in readability. LLMs may have a greater application in oncology than the current literature suggests. Future studies might investigate the generalizability of contextualized LLMs to other malignancies, prospective trials studying model efficacy within real-world applications, and whether the utilized guidelines might produce biases depending on the cancer subtype. While this study provides proof of concept for the future investigation of contextualized GPTs in oncology and outlines key elements of their design, any clinical deployment will require validation in prospective trials and FDA approval as a medical device.

## Appendices

Appendix A: Written instructions

**Table 3:** Prompt and PDF+Prompt Written Instructions Written instructions were injected into the Prompt and Prompt+PDF models as shown.

Prompt	Prompt + PDF
You are a prominent Molecular Tumor Board specialized in the treatment of Non-Small Cell Lung Cancer (NSCLC) and its subtypes. Patients diagnosed with advanced or metastatic NSCLC are often referred to you by oncologists for biomarker-directed and standard of care therapy. After reading a patient vignette, you make sure to refer to the latest guidelines before recommending therapies which align with NCCN or ASCO recommendations for the patient's clinical context. The sources you pay particularly close attention to include: Non–Small Cell Lung Cancer, Version 6.2024, NCCN Clinical Practice Guidelines in Oncology Therapy for Stage IV Non–Small Cell Lung Cancer With Driver Alterations: ASCO Living Guideline, Version 2023.3 You will structure your first choice recommended therapy as: - **Drug Name** - **Evidence Supporting the Recommendation** - **Citation**: You excel at providing concise therapy recommendations which parallel clinical guidelines, emphasize preferred therapies, and do not include redundant or inferior treatments.	You are a prominent Molecular Tumor Board specialized in the treatment of Non-Small Cell Lung Cancer (NSCLC) and its subtypes. Patients diagnosed with advanced or metastatic NSCLC are often referred to you by oncologists for biomarker-directed and standard of care therapy. After reading a patient vignette, you make sure to refer to the latest guidelines before recommending therapies which align with NCCN or ASCO recommendations for the patient's clinical context. The sources you pay particularly close attention to include: NCCN NCSL 2024 Guidelines.pdf ASCO With Driver 2024 Guidelines.pdf You will structure your first choice recommended therapy as: - **Drug Name** - **Evidence Supporting the Recommendation** - **Citation**: You excel at providing concise therapy recommendations which parallel clinical guidelines, emphasize preferred therapies, and do not include redundant or inferior treatments.

Appendix B: Questions and responses: Expert consensus

**Table 4:** Questions and Responses: Expert Consensus Expert consensuses, modified questions, and ChatGPT responses are numerically ordered by NCCN guideline to allow for cross referencing across tables. Relevant preferred or category 1 therapies used for grading are bolded.

Q#	NCCN Reccomendation
NSCL-21	For EGFR EXON 19 DELETION OR EXON 21 L858R MUTATIONS discovered prior to first-line systemic therapy: Preferred Osimertinib (category 1) Other Recommended Osimertinib + pemetrexed + (cisplatin or carboplatin) (nonsquamous) (category 1) or Erlotinib (category 1) or Afatinib (category 1) or Gefitinib (category 1) or Dacomitinib (category 1) or Erlotinib + ramucirumab or Erlotinib + bevacizumab
NCSL-22	For EGFR EXON 19 DELETION OR EXON 21 L858R MUTATIONS with progression on osimertinib which is symptomatic, systemic, and contains multiple lesions: Amivantamab-vmjw + carboplatin + pemetrexed (nonsquamous) (category 1) (Preferred) or Systemic therapy, Adenocarcinoma (NSCL-K 1 of 5) or Squamous Cell Carcinoma (NSCL-K 2 of 5)
NCSL-23	For EGFR EXON 19 DELETION OR EXON 21 L858R MUTATIONS with progression on erlotinib (± ramucirumab or bevacizumab), afatinib, gefitinib, or dacomitinib: • Consider definitive local therapy (eg, SABR or surgery) for limited lesions • Osimertinib (if T790M+) (category 1) or Continue erlotinib (± ramucirumab or bevacizumabss) or afatinib or gefitinib or dacomitinib (if T790M-)
NSCL-24	For EGFR S768I, L861Q, and/ or G719X mutations discovered prior to first-line systemic therapy: Preferred Afatinib or Osimertinib Other Recommended Erlotinib or Gefitinib or Dacomitinib
NSCL-25	For EGFR EXON 20 INSERTION MUTATION: Amivantamab-vmjw + carboplatin/ pemetrexed (nonsquamous) (category 1) (Preferred) or Systemic therapy Adenocarcinoma (NSCL-K 1 of 5) or Squamous Cell Carcinoma (NSCL-K 2 of 5)
NSCL-27	For ALK rerrangment discovered prior to first-line systemic therapy: Preferred Alectinib (category 1) or Brigatinib (category 1) or Lorlatinib (category 1) Other Recommended Ceritinib (category 1) Useful in Certain Circumstances Crizotinib (category 1)
NSCL-30	For ROS1 rearrangment discovered prior to first-line systemic therapy: Preferred Entrectinib or Crizotinib or Repotrectinib or Other Recommended Ceritinib
NSCL-32	For BRAF V600E mutation discovered prior to first-line systemic therapy: Preferred Dabrafenib + trametinib or Encorafenib + binimetinib Useful in Certain Circumstances Vemurafenib or dabrafenib Other Recommended Systemic Therapy for Adenocarcinoma (NSCL-K 1 of 5) or Squamous Cell C
NSCL-33	For NTRK1/2/3 gene fusion discovered prior to first line systemic therapy: Preferred Larotrectinib or Entrectinib or Repotrectinib Useful in Certain Circumstances Systemic Therapy Adenocarcinoma (NSCL-K 1 of 5) or Squamous Cell Carcinoma (NSCL-K 2 of 5)
NSCL-34	For METex14 skipping mutation discovered prior to first-line systemic therapy: Preferred Capmatinib or Tepotinib Useful in Certain Circumstances Crizotinib or Systemic Therapy Adenocarcinoma (NSCL-K 1 of 5) or Squamous Cell Carcinoma (NSCL-K 2 of 5)
NSCL-35	For RET rearrangement discovered prior to first-line systemic therapy: Preferred Selpercatinib or Pralsetinib Useful in Certain Circumstances Cabozantinib Other Recommended Systemic Therapy Adenocarcinoma (NSCL-K 1 of 5) or Squamous Cell Carcinoma (NSCL-K 2 of 5)
NSCL-36	For patients with ERBB2 (HER2) MUTATION who demonstrate progression following systemic therapy with carboplatin: Preferred Fam-trastuzumab deruxtecan-nxki or Other Recommended Ado-trastuzumab emtansine

Appendix C: Questions and responses: Modified questions

**Table 5:** Modified Questions

Q#	Modified Question
NSCL-21	Given an advanced lung adenocarcinoma patient with EGFR p. L858R mutations. What therapy or therapies would be preferred?
NCSL-22	Given an advanced lung adenocarcinoma patient with EGFR p. L858R mutations, demonstrating progression on osimertinib and multiple metastases. What therapy or therapies would be preferred?
NCSL-23	Given an advanced lung adenocarcinoma patient with progression on aftatinib and EGFR p. L858R, T790M mutations. What therapy or therapies would be preferred?
NSCL-24	Given an advanced lung adenocarcinoma patient with G719X mutations. What therapy or therapies would be preferred?
NSCL-25	Given an advanced lung adenocarcinoma patient with EGFR Exon 20 Asp770_Asn771ins mutations. What therapy or therapies would be preferred?
NSCL-27	Given an advanced lung adenocarcinoma patient with EML4-ALK fusion mutations. What therapy or therapies would be preferred?
NSCL-30	Given an advanced lung adenocarcinoma patient with no prior treatment and CD74-ROS1 fusion mutations. What therapy or therapies would be preferred?
NSCL-32	Given an advanced lung adenocarcinoma patient with no prior treatment and BRAF V600E fusion mutations. What therapy or therapies would be preferred?
NSCL-33	Given an advanced lung adenocarcinoma patient with ETV6-NTRK3 mutations. What therapy or therapies would be preferred?
NSCL-34	Given an advanced lung adenocarcinoma patient with MET c.3028 + 2T>C mutations. What therapy or therapies would be preferred?
NSCL-35	Given an advanced lung adenocarcinoma patient with CCDC6::RET gene fusion mutations. What therapy or therapies would be preferred?
NSCL-36	Given an advanced lung adenocarcinoma patient with ERBB2 A775_G776insYVMA mutations, demonstrating recurrence after treatment with cisplatin and pemetrexed. What therapy or therapies would be preferred?

Appendix D: Questions and responses: GPT-4

**Table 6:** GPT4 Responses

Q#	ChatGPT4 Response
NSCL-21	For patients with advanced lung adenocarcinoma who have an EGFR exon 21 L858R mutation, targeted therapy with tyrosine kinase inhibitors (TKIs) is typically recommended. Here are some of the specific treatment options often considered: First-Generation EGFR TKIs: These include drugs like erlotinib and gefitinib. These have been widely used as first-line treatments in patients with EGFR mutations, including the L858R mutation. They target the ATP binding site of the EGFR tyrosine kinase, which is critical for the cancer cell's growth and survival. Second-Generation EGFR TKIs: Afatinib and dacomitinib fall into this category. They offer a broader inhibition of the ErbB family of receptors and may be used as initial treatment in some settings. These drugs tend to have a slightly different side effect profile and may be more potent but can also have increased toxicity. Third-Generation EGFR TKIs: Osimertinib is a more recent addition, highly effective for patients with EGFR T790M mutations, which often develop as resistance mechanisms to first- and second-generation TKIs. However, it's also increasingly used as a first-line treatment due to its effectiveness and better penetration of the blood-brain barrier, which is beneficial for patients with or at risk of brain metastases. Combination Therapies: Research is ongoing into the benefits of combining EGFR TKIs with other therapies, including chemotherapy, angiogenesis inhibitors (like bevacizumab), and potentially immunotherapy, though the latter may be less effective in EGFR-mutant lung cancer compared to other types of non-small cell lung cancer. The choice of specific therapy often depends on several factors including the patient's overall health, presence of other mutations or health conditions, potential side effects, and prior treatments. Decisions are typically made by an oncologist specializing in lung cancer, often in consultation with a multidisciplinary team. Furthermore, clinical trials might offer access to newer therapies and combinations that are not yet widely available but could be beneficial depending on the individual patient’s condition.
NCSL-22	For a patient with advanced lung adenocarcinoma harboring EGFR p.L858R mutations, showing progression on osimertinib with multiple metastases, the treatment approach needs to consider several factors including the specific mechanisms of resistance to osimertinib, the patient’s performance status, previous treatments, and the presence of specific genomic alterations. Here are some potential strategies: Biopsy and Molecular Testing: It's crucial to identify the mechanism of resistance to osimertinib. A repeat biopsy and comprehensive genomic profiling can reveal mutations or alterations such as C797S mutation, MET amplification, or other bypass track activations, which can guide further treatment. Liquid biopsy might also be considered if a tissue biopsy is not feasible. Combination Therapies: Based on the results of the resistance mechanisms, combination therapies can be considered. For example: If MET amplification is detected, combining a MET inhibitor (like capmatinib or crizotinib) with osimertinib might be effective. For patients with a C797S mutation, a combination of a first or second-generation EGFR TKI (like erlotinib or afatinib) with osimertinib could be considered. Chemotherapy: If targeted therapy options are not viable or if the cancer has a broad resistance profile, systemic chemotherapy could be the next step. Options typically include pemetrexed-based regimens, possibly combined with platinum agents, depending on what the patient has previously received. Clinical Trials: Enrolling in a clinical trial could provide access to novel therapies or combinations that are not available otherwise. Trials may focus on new EGFR inhibitors, combination therapies targeting resistance mechanisms, or emerging treatments like antibody-drug conjugates or immune therapies tailored to specific genetic alterations. Supportive Care and Local Treatments: In addition to systemic therapy, supportive care to manage symptoms and improve quality of life is essential. Palliative radiotherapy might be considered for symptom control or to manage specific sites of painful or life-threatening metastases. Each of these options should be discussed with a multidisciplinary team to tailor the approach based on the specific clinical situation of the patient. The choice of therapy often involves balancing the potential benefits with the side effects and the patient’s preferences and overall health status.
NCSL-23	For a patient with advanced lung adenocarcinoma who has shown progression on afatinib and has EGFR mutations p.L858R and T790M, the preferred treatment option is typically a third-generation EGFR tyrosine kinase inhibitor (TKI). Osimertinib is specifically designed to target both the initial activating EGFR mutations (like L858R) and the resistant T790M mutation. It has shown significant efficacy in this patient population. Osimertinib is approved for the treatment of patients with metastatic EGFR T790M mutation-positive non-small cell lung cancer (NSCLC) who have progressed on or after EGFR TKI therapy. This recommendation is based on clinical trials that have demonstrated osimertinib’s ability to improve progression-free survival and overall survival in this specific group of patients. Additionally, it would be prudent to conduct comprehensive genomic profiling at the time of progression to identify any other potential actionable mutations or alterations that could influence therapy choices. This can include testing for other resistance mechanisms or mutations in genes such as MET, HER2, or BRAF, which might open up additional targeted therapy options or eligibility for clinical trials. Furthermore, discussing the case in a multidisciplinary team setting and considering the patient’s overall health status, previous treatments, and potential side effects is crucial in making an individualized treatment plan. If osimertinib is not suitable, or if further progression occurs, options may include chemotherapy, immunotherapy, or participation in clinical trials exploring novel agents or combinations. It’s also recommended to closely monitor the patient for side effects and to manage these proactively to improve the quality of life and treatment adherence.
NSCL-24	In the case of a lung adenocarcinoma patient with G719X mutations, targeted therapy options are particularly important to consider. The G719X mutation is a specific type of mutation in the epidermal growth factor receptor (EGFR) gene, which can be effectively targeted with certain tyrosine kinase inhibitors (TKIs). Here are the preferred therapies based on current guidelines and clinical evidence: Osimertinib: This is a third-generation EGFR tyrosine kinase inhibitor that is highly effective against tumors with EGFR mutations, including G719X. Osimertinib is generally well-tolerated and has shown high efficacy in various clinical trials. It is often used as a first-line treatment for EGFR-mutant non-small cell lung cancer (NSCLC). Other EGFR TKIs: First- and second-generation EGFR TKIs like erlotinib, gefitinib, and afatinib may also be considered. These agents have been used effectively in treating NSCLC with EGFR mutations. Among these, afatinib has been specifically studied and shown efficacy in treating patients with uncommon EGFR mutations including G719X. Combination Therapies and Clinical Trials: For some patients, especially those who progress on initial therapy or have additional complicating factors, participation in clinical trials may be advisable. Trials might offer access to newer therapies or combinations of therapies that could be more effective based on the tumor’s genetic profile and previous treatment responses. The choice of therapy should be guided by a thorough evaluation of the patient’s overall health, specific characteristics of the cancer, potential side effects, and the presence of any other mutations that might affect treatment efficacy. Regular monitoring and follow-up are essential to adjust the treatment plan as needed based on the patient’s response and any side effects experienced.
NSCL-25	The treatment of advanced lung adenocarcinoma with EGFR Exon 20 insertion mutations involves specific targeted therapies. These mutations are somewhat less common and can be more challenging to treat than the more typical EGFR mutations like Exon 19 deletions or the L858R mutation. However, recent developments have led to new options specifically targeting Exon 20 insertions. For patients with EGFR Exon 20 insertion mutations in non-small cell lung cancer (NSCLC), the following therapies are commonly considered: Amivantamab (Rybrevant): This is a bispecific antibody targeting EGFR and MET receptors, approved by the FDA specifically for the treatment of adult patients with locally advanced or metastatic NSCLC with EGFR Exon 20 insertion mutations. Amivantamab has shown effectiveness in shrinking tumors and managing disease progression in this particular subgroup of patients. Mobocertinib (Exkivity): Mobocertinib is an oral tyrosine kinase inhibitor (TKI) that specifically targets EGFR Exon 20 insertion mutations. It has been approved by the FDA for patients with metastatic NSCLC harboring these mutations who have received prior platinum-based chemotherapy. These therapies represent the most targeted and recent advancements for treating this specific mutation in lung adenocarcinoma. In practice, the choice between these options might depend on the patient's overall health, prior treatments, potential side effects, and specific characteristics of the mutation. It is also important to discuss the case in a multidisciplinary team and consider clinical trials, as ongoing research may provide access to emerging therapies or novel combinations that could be beneficial. In addition to targeted therapy, the management plan may include other supportive care measures and treatments depending on the patient's general condition and the presence of symptoms or complications from the disease.
NSCL-27	For patients with advanced lung adenocarcinoma who have an EML4-ALK fusion mutation, targeted therapy using ALK inhibitors is typically the preferred treatment approach. This mutation leads to the expression of an abnormal ALK protein that drives cancer cell growth, and ALK inhibitors can specifically target and block the activity of this protein. Here are some of the common ALK inhibitors used: Crizotinib: This was the first ALK inhibitor approved by the FDA and is often used as a first-line treatment for ALK-positive non-small cell lung cancer (NSCLC). Ceritinib: Approved for use in patients who have become resistant to crizotinib or as a first-line treatment. Alectinib: It has shown high efficacy and is often used as a first-line therapy due to its ability to also effectively treat or prevent brain metastases, which are common in ALK-positive NSCLC. Brigatinib: Also used after crizotinib resistance, or as a first-line treatment. It has shown potent activity against ALK mutations and good control of brain metastases. Lorlatinib: A newer ALK inhibitor that is effective against several known ALK mutations that confer resistance to earlier-generation ALK inhibitors. It is typically used after other ALK inhibitors have failed. The choice of specific ALK inhibitor can depend on several factors, including: The patient's overall health and presence of other medical conditions. Potential side effects of the drug. Previous treatments the patient has undergone. The presence of brain metastases. Resistance mutations in the ALK gene. Ongoing clinical trials continue to evaluate the efficacy of existing ALK inhibitors and explore new therapeutic options, which could potentially offer better outcomes and manage resistance mechanisms. Regular testing and monitoring for resistance mutations can guide adjustments in therapy to maintain control of the disease. It is important for treatment to be guided by a medical oncologist who specializes in lung cancer, and who can provide personalized treatment plans based on the most up-to-date research and clinical guidelines.
NSCL-30	For an advanced lung adenocarcinoma patient with the CD74-ROS1 fusion mutation who has not previously been treated, the preferred therapeutic approach typically centers on targeted therapy using ROS1 inhibitors. ROS1 is a receptor tyrosine kinase, and mutations involving ROS1 fusions can drive cancer development by activating signaling pathways that promote cell growth and survival. Here are some key points and treatment options: Crizotinib: This is an oral small-molecule inhibitor that targets ROS1 and has been shown to be effective in treating ROS1-positive non-small cell lung cancer (NSCLC). It was the first ROS1 inhibitor approved by the FDA for this purpose based on the results of clinical trials demonstrating significant tumor response rates and progression-free survival benefits. Other ROS1 Inhibitors: Additional targeted therapies for ROS1-positive NSCLC include: Entrectinib: This drug is a newer generation inhibitor that also targets NTRK and ALK in addition to ROS1. It is particularly noted for its ability to penetrate the central nervous system, making it useful in cases where there is brain involvement. Lorlatinib: Another option for patients, particularly those who may have developed resistance to earlier treatments like crizotinib. Lorlatinib is a third-generation inhibitor that targets ALK and ROS1 and has shown activity against brain metastases. Monitoring and Management of Resistance: While ROS1 inhibitors can be highly effective, resistance to therapy often develops. It is important to monitor the patient’s response and consider biopsies or liquid biopsies to detect resistance mutations. This can guide the choice of subsequent lines of therapy, including switching to different ROS1 inhibitors or combining with other treatments. Clinical Trials: Given the rapidly evolving landscape of targeted therapies, participation in clinical trials can be considered to access novel therapeutic options that might be beneficial. Comprehensive Care: Beyond targeted therapies, the management of an advanced lung cancer patient should include supportive care to manage symptoms, improve quality of life, and address any complications of the disease or its treatment. It is crucial for treatment to be guided by an oncologist specialized in lung cancer, who can tailor therapy choices to the specific genetic profile and overall health status of the patient. Regular follow-ups and adjustments in the treatment plan based on the response to therapy and evolving clinical evidence are key components of effective management.
NSCL-32	For a patient with advanced lung adenocarcinoma harboring a BRAF V600E mutation, specific targeted therapies are recommended based on current clinical guidelines and research findings. The BRAF V600E mutation is a specific genetic alteration that can be effectively targeted by BRAF inhibitors, which are drugs designed to directly inhibit the activity of the mutated BRAF protein. The standard therapy for lung adenocarcinoma patients with this mutation typically involves: BRAF Inhibitors: These drugs specifically target the BRAF V600E mutation. Vemurafenib and dabrafenib are examples of BRAF inhibitors that have shown efficacy in treating various cancers with BRAF mutations, including melanoma and lung cancer. Combination Therapy with MEK Inhibitors: Combining BRAF inhibitors with MEK inhibitors (another type of targeted therapy) has been found to enhance treatment efficacy and overcome resistance that might develop with BRAF inhibitor monotherapy. For instance, the combination of dabrafenib (a BRAF inhibitor) and trametinib (a MEK inhibitor) has been approved for use in non-small cell lung cancer (NSCLC) with BRAF V600E mutations. This combination has been shown to improve survival rates and reduce the risk of disease progression compared to treatment with BRAF inhibitors alone. Clinical trials have supported the use of these combinations, demonstrating significant benefits in terms of response rate and progression-free survival. It's crucial for treatment decisions to be based on a comprehensive evaluation by an oncologist, who can consider the patient’s overall health, the extent of the disease, potential side effects, and the latest clinical guidelines and research data. Additionally, supportive care to manage symptoms and improve quality of life is vital in the treatment plan for advanced lung adenocarcinoma patients.
NSCL-33	The presence of the ETV6-NTRK3 fusion gene in a patient with advanced lung adenocarcinoma suggests that targeted therapy using TRK inhibitors could be particularly effective. The ETV6-NTRK3 gene fusion results in the expression of a chimeric tropomyosin receptor kinase (TRK) protein, which can be specifically targeted by TRK inhibitors. Here’s a breakdown of the relevant therapies: TRK Inhibitors: Drugs like larotrectinib (Vitrakvi) and entrectinib (Rozlytrek) are FDA-approved TRK inhibitors that have shown high efficacy in treating tumors harboring NTRK gene fusions, including those in lung cancer. These drugs work by specifically targeting and inhibiting the activity of TRK fusion proteins, which drive the growth and survival of cancer cells. Clinical Trials: For patients with this specific mutation, participation in clinical trials can be considered if standard therapies do not yield satisfactory results or if the patient is eligible for trials exploring new therapies or combinations of existing therapies. Comprehensive Genomic Profiling: It's also important to perform comprehensive genomic profiling of the tumor to identify any other potential actionable mutations that might inform additional targeted therapy options or combinations. Other Therapies: Depending on the patient's overall health, extent of disease, and previous treatments, other standard treatments for advanced lung adenocarcinoma—such as chemotherapy, immunotherapy, or a combination thereof—might also be considered, often in conjunction with targeted therapies. Deciding on the best course of action should involve a detailed discussion with a multidisciplinary oncology team, taking into account the patient's overall condition, prior treatment history, and specific genetic findings. Always, the patient's preferences and quality of life considerations should play a central role in choosing the therapy.
NSCL-34	For patients with advanced lung adenocarcinoma harboring specific MET mutations, treatment approaches can vary based on the type of mutation and the evidence supporting the efficacy of certain therapies. The MET c.3028+2T>C mutation is a splice-site mutation, which could potentially affect the normal splicing of the MET gene transcript, thereby altering the function of the MET protein. Such mutations can lead to aberrant activation of the MET signaling pathway, which is implicated in tumor growth and survival. The treatment options might include the following: MET Inhibitors: Targeted therapies with MET inhibitors could be beneficial. Currently, there are several MET inhibitors that have shown promise in clinical trials for treating MET-altered lung cancers, including: Capmatinib (Tabrecta): Approved by the FDA for metastatic non-small cell lung cancer (NSCLC) with MET exon 14 skipping mutations. Although primarily indicated for exon 14 skipping mutations, it might also show activity in other MET dysregulations. Tepotinib: Another MET inhibitor approved for MET exon 14 skipping mutations in NSCLC. Similar to capmatinib, its efficacy could potentially extend to other MET-related alterations depending on the specific mutation characteristics and clinical evidence. Clinical Trials: Given the specific and less common nature of the MET c.3028+2T>C mutation, participation in clinical trials could be a valuable option to access novel therapies or new uses of existing drugs that are not yet approved for this specific mutation. Comprehensive Genomic Profiling: It's crucial to perform comprehensive genomic profiling of the tumor to identify any other actionable mutations or alterations that could influence treatment decisions. This may open up additional targeted therapy options or combination therapies based on the tumor’s genomic landscape. Chemotherapy and Immunotherapy: If targeted therapy is not suitable or if the disease progresses on targeted treatments, standard treatment options such as chemotherapy and immunotherapy may be considered, depending on the patient’s overall health, prior treatments, and specific clinical factors. It is essential for treatment decisions to be made in a multidisciplinary setting, incorporating the insights from oncologists, pathologists, and possibly genetic counselors. This ensures that all aspects of the patient's disease and personal health are considered to optimize treatment outcomes. Furthermore, ongoing monitoring and re-evaluation of the disease response to treatment are important to adjust the therapy plan as needed.
NSCL-35	For a patient with advanced lung adenocarcinoma featuring a CCDC6-RET gene fusion, targeted therapy specifically designed to inhibit the RET protein would be the preferred treatment option. RET gene fusions are a known driver mutation in non-small cell lung cancer (NSCLC), including adenocarcinomas, and can be effectively targeted by certain drugs. As of my last update, several RET-specific inhibitors have shown promise and have been approved by regulatory agencies like the FDA: Selpercatinib (Retevmo): This drug is a selective RET kinase inhibitor that has been approved for the treatment of adult patients with metastatic RET fusion-positive NSCLC. Clinical trials have demonstrated significant efficacy in shrinking tumors and in some cases, prolonged disease control. Pralsetinib (Gavreto): Similar to selpercatinib, pralsetinib is another RET inhibitor that has been approved for use in patients with RET fusion-positive NSCLC. It offers another effective option for targeting tumors driven by RET alterations. Both of these drugs are typically considered when a RET fusion is identified, given their targeted mechanism of action which can lead to better outcomes and potentially fewer side effects compared to conventional chemotherapy. The choice between these therapies may depend on specific patient factors including the overall health of the patient, presence of any other mutations or health conditions, prior treatments, and potentially the specific characteristics of the RET fusion. Moreover, the choice might be influenced by the availability of the drug, insurance coverage, and patient or physician preference. Continual monitoring and testing are crucial as resistance to these therapies can develop, and adjustments to the treatment regimen may be necessary over time. Also, it’s important for the medical team to manage any side effects associated with these therapies to ensure the best possible quality of life for the patient.
NSCL-36	The treatment for a lung adenocarcinoma patient with an ERBB2 (also known as HER2) A775_G776insYVMA mutation who has shown recurrence after initial treatment with cisplatin and pemetrexed can be complex and should be guided by updated clinical data and trials, along with the advice of a multidisciplinary oncology team. The A775_G776insYVMA is a specific insertion mutation in the HER2 gene that can affect treatment response and options. Here are some considerations and potential therapies: 1. HER2-Targeted Therapy: Patients with HER2 mutations may benefit from HER2-targeted therapies. Drugs such as trastuzumab (an anti-HER2 monoclonal antibody), often used in breast cancer, have been studied in lung cancers with HER2 mutations. However, the efficacy in lung cancer, especially with specific mutations like A775_G776insYVMA, may vary and is still under investigation. 2. Tyrosine Kinase Inhibitors (TKIs): Small molecule TKIs that target HER2, such as afatinib, have shown some promise in non-small cell lung cancer (NSCLC) with HER2 mutations. These drugs inhibit the kinase activity of the HER2 protein, which can help control tumor growth. 3. Clinical Trials: Given the recurrent nature of the disease and the specific mutation, participation in clinical trials may be an optimal approach to access novel therapies and regimens that are not yet widely available. These might include newer HER2 inhibitors or combination therapies targeting multiple pathways. 4. Chemotherapy and Immunotherapy: Depending on the patient’s overall health, performance status, and previous treatment history, further chemotherapy or immunotherapy might be options. Drugs such as docetaxel (a chemotherapy agent) or pembrolizumab (a PD-1 inhibitor immunotherapy) could be considered, particularly if there is no specific targeted therapy available. 5. Comprehensive Genomic Profiling: It’s also advisable to perform comprehensive genomic profiling of the tumor if not already done. This can help identify additional actionable mutations or fusions that may be targeted with specific therapies. 6. Palliative Care: For managing symptoms and maintaining quality of life, palliative care should be integrated into the treatment plan, especially in cases of advanced disease. The choice of therapy should be personalized based on the detailed genetic makeup of the tumor, the patient’s prior treatment history, and overall health. Consultation with a specialist in thoracic oncology who is knowledgeable about the latest treatments and research is crucial.

Appendix E: Questions and responses: GPT-4o

**Table 7:** GPT4o Responses

Q#	ChatGPT4o Response
NSCL-21	For an advanced lung adenocarcinoma patient with EGFR p. L858R mutations, the preferred therapy is typically a targeted therapy using an EGFR tyrosine kinase inhibitor (TKI). The choice of EGFR TKIs includes: First-line therapy options: Osimertinib (Tagrisso): This is often the preferred first-line treatment due to its efficacy and favorable side effect profile. Osimertinib has shown superior overall survival and progression-free survival compared to first- and second-generation TKIs in patients with EGFR mutations. Erlotinib (Tarceva), Gefitinib (Iressa), and Afatinib (Gilotrif): These are first- and second-generation TKIs that were previously standard first-line treatments. While still effective, they are generally considered less favorable compared to osimertinib due to a higher risk of certain side effects and lower overall efficacy. Subsequent therapy options if resistance develops or if there is disease progression: If the patient initially receives a first- or second-generation TKI and develops resistance due to a secondary EGFR T790M mutation, Osimertinib can be used as a second-line therapy. In the absence of T790M mutation, or after progression on osimertinib, subsequent treatment options may include chemotherapy, other targeted therapies based on additional molecular profiling, or inclusion in clinical trials. Combination therapies: Recent studies and ongoing clinical trials are exploring the combination of EGFR TKIs with other agents like anti-angiogenic drugs (e.g., bevacizumab) or immune checkpoint inhibitors. However, these combinations are not yet standard and should be considered based on individual patient factors and availability of clinical trials. Summary For an advanced lung adenocarcinoma patient with EGFR p. L858R mutations, Osimertinib is currently the preferred first-line therapy due to its efficacy and safety profile. If resistance develops, especially due to T790M mutation, osimertinib remains a viable second-line option. Otherwise, further treatment decisions should be guided by additional molecular testing and patient-specific factors.
NCSL-22	For a patient with advanced lung adenocarcinoma with EGFR p. L858R mutation who has progressed on osimertinib and has multiple metastases, the following therapeutic options can be considered: Combination Chemotherapy: Platinum-based doublet chemotherapy (e.g., carboplatin or cisplatin combined with pemetrexed) is often considered standard treatment after progression on EGFR TKIs like osimertinib. Immunotherapy: Immune checkpoint inhibitors such as pembrolizumab, nivolumab, or atezolizumab can be considered, especially if the tumor expresses PD-L1. However, the efficacy of immunotherapy in EGFR-mutant lung cancers has been generally lower compared to EGFR wild-type cancers. Combination of Chemotherapy and Immunotherapy: Combining chemotherapy with immunotherapy, such as pembrolizumab plus pemetrexed and platinum-based chemotherapy, may be considered based on the patient's overall health and PD-L1 status. Clinical Trials: Enrolling in clinical trials can provide access to novel therapies, including new targeted therapies, combination regimens, or next-generation EGFR TKIs. For instance, trials involving drugs targeting MET amplification or other resistance mechanisms might be relevant. Targeted Therapies for Resistance Mechanisms: If specific resistance mechanisms are identified (e.g., MET amplification, HER2 mutations, or BRAF mutations), targeted therapies against those alterations might be beneficial. This requires molecular testing to identify these mechanisms. Local Therapy: For patients with oligometastatic disease (limited number of metastases), local treatments such as stereotactic body radiation therapy (SBRT) or surgery might be considered for disease control. Supportive Care and Symptom Management: In cases where further aggressive therapy is not suitable, focusing on symptom management and improving quality of life through palliative care is important. It is crucial to personalize the treatment plan based on the patient's overall health, performance status, specific resistance mechanisms, and preferences. Multidisciplinary discussions involving oncologists, radiologists, and other healthcare professionals are often necessary to determine the best approach.
NCSL-23	For a patient with advanced lung adenocarcinoma who has the EGFR p.L858R and T790M mutations and has progressed on afatinib, the next line of therapy typically involves a third-generation EGFR tyrosine kinase inhibitor (TKI). The preferred therapy in this case is: Osimertinib (Tagrisso): This third-generation EGFR TKI is specifically designed to target both the EGFR T790M resistance mutation and the common activating mutations such as p.L858R. Osimertinib has shown significant efficacy in patients with the T790M mutation and is often the treatment of choice in this scenario. Additional Considerations: Clinical Trials: Participation in clinical trials can provide access to new therapies and combinations that might be beneficial, especially if standard options are exhausted. Combination Therapies: Emerging evidence suggests potential benefits from combining EGFR TKIs with other treatments such as immune checkpoint inhibitors, anti-angiogenic agents, or other targeted therapies. However, these approaches are generally considered within clinical trials or specific clinical contexts. Systemic Chemotherapy: If the patient progresses on osimertinib or if osimertinib is not suitable, standard chemotherapy regimens such as pemetrexed and platinum-based doublet chemotherapy might be considered. Local Therapies: For oligoprogression (progression limited to a few sites), local treatments such as radiotherapy or surgery combined with continuation of osimertinib may be considered. The choice of therapy should be personalized based on the patient's overall health, prior treatments, specific genetic mutations, and other factors. Regular monitoring and assessment by an oncologist specializing in lung cancer are essential to optimize treatment outcomes.
NSCL-24	For a patient with advanced lung adenocarcinoma harboring G719X mutations, the preferred therapies typically involve targeted treatments, specifically those that target EGFR (epidermal growth factor receptor) mutations. G719X mutations are less common but still significant EGFR mutations that can respond to EGFR tyrosine kinase inhibitors (TKIs). The preferred therapies include: First-Generation EGFR TKIs: Gefitinib Erlotinib These drugs have shown efficacy in patients with uncommon EGFR mutations, including G719X, though they might have varying degrees of effectiveness compared to more common mutations. Second-Generation EGFR TKI: Afatinib: Clinical data suggest that afatinib can be effective in patients with G719X mutations. It has been shown to provide a more comprehensive inhibition of the EGFR family and can be effective against a broader spectrum of EGFR mutations. Third-Generation EGFR TKI: Osimertinib: While primarily used for T790M-positive EGFR mutations, osimertinib has shown some activity against a variety of uncommon EGFR mutations, though specific data for G719X mutations might be limited. In some cases, combination therapies or clinical trials involving novel agents may be considered, especially if the patient has progressed on standard EGFR TKIs. It is also essential for the treating oncologist to consider the overall clinical condition of the patient, potential side effects, and other comorbidities when choosing the appropriate therapy. Molecular testing and a multidisciplinary approach can further help in tailoring the best treatment strategy for the patient.
NSCL-25	For a patient with advanced lung adenocarcinoma harboring EGFR exon 20 insertions, including the specific Asp770_Asn771ins mutation, the treatment landscape is evolving, but there are several preferred therapies based on the latest clinical guidelines and research. Mobocertinib (Exkivity): This is an oral tyrosine kinase inhibitor specifically approved by the FDA for patients with EGFR exon 20 insertion mutations in advanced non-small cell lung cancer (NSCLC) who have progressed on or after platinum-based chemotherapy. Mobocertinib targets these specific mutations and has shown efficacy in clinical trials. Amivantamab-vmjw (Rybrevant): This is an intravenous bispecific antibody that targets EGFR and MET. It is also approved by the FDA for the treatment of patients with NSCLC with EGFR exon 20 insertion mutations who have disease progression on or after platinum-based chemotherapy. Platinum-Based Chemotherapy: Traditionally, patients with advanced lung adenocarcinoma, including those with EGFR exon 20 insertions, might be treated initially with platinum-based chemotherapy regimens (such as cisplatin or carboplatin combined with pemetrexed). This approach can still be used, especially in patients who have not received prior chemotherapy. Clinical Trials: Participation in clinical trials can provide access to emerging therapies and novel targeted treatments that may be effective against EGFR exon 20 insertion mutations. Ongoing research and clinical trials are essential for discovering new treatments and combinations. Immune Checkpoint Inhibitors: While generally less effective as monotherapy in patients with EGFR mutations, immune checkpoint inhibitors like pembrolizumab (Keytruda) or nivolumab (Opdivo) may be considered in combination with chemotherapy or in patients with high PD-L1 expression after other options have been exhausted. It is important for the treating oncologist to consider the patient's overall health, prior treatments, and specific mutation characteristics when selecting the most appropriate therapy. Consulting with a specialized cancer center or a molecular tumor board can also help in tailoring the best treatment approach for the individual patient.
NSCL-27	For a patient with advanced lung adenocarcinoma harboring an EML4-ALK fusion mutation, the preferred therapies would typically be ALK inhibitors. These targeted therapies are designed to specifically inhibit the activity of the ALK fusion protein, which drives cancer cell growth. The following ALK inhibitors are commonly used: Alectinib (Alecensa): Often preferred as the first-line treatment due to its efficacy and favorable side effect profile. Brigatinib (Alunbrig): Another effective first-line treatment option. Ceritinib (Zykadia): Can be used as a first-line treatment or for patients who have progressed on crizotinib. Crizotinib (Xalkori): The first ALK inhibitor approved, but now often used when other therapies are not available or suitable. Lorlatinib (Lorbrena): Typically used for patients who have progressed on other ALK inhibitors. The choice among these therapies can depend on various factors, including the patient's overall health, presence of brain metastases, and prior treatments. Alectinib and brigatinib are frequently preferred due to their higher efficacy and better central nervous system penetration compared to crizotinib. It's essential for treatment to be personalized, considering potential side effects and the patient's overall condition. Consultation with a medical oncologist specializing in lung cancer is crucial to determine the most appropriate therapy.
NSCL-30	For a patient with advanced lung adenocarcinoma harboring a CD74-ROS1 fusion mutation and no prior treatment, the preferred therapies are typically targeted therapies that specifically inhibit ROS1. The following therapies are recommended based on current clinical guidelines and evidence: Crizotinib: This is often the first-line treatment for patients with ROS1-positive lung adenocarcinoma. Crizotinib is a tyrosine kinase inhibitor (TKI) that targets ALK, ROS1, and MET. It has shown significant efficacy in patients with ROS1 rearrangements. Entrectinib: Another targeted therapy option, entrectinib is also a TKI that inhibits ROS1, TRK, and ALK. It is approved for the treatment of ROS1-positive non-small cell lung cancer (NSCLC) and has shown efficacy in both systemic disease and brain metastases. Lorlatinib: This TKI is primarily known for its use in ALK-positive NSCLC, but it is also effective against ROS1-positive lung cancer. Lorlatinib can be considered especially in cases where the cancer has progressed on crizotinib, due to its ability to penetrate the blood-brain barrier and its activity against certain ROS1 resistance mutations. In addition to these targeted therapies, comprehensive genomic profiling should be considered to identify any other potential mutations or alterations that could influence treatment decisions. Close monitoring for response to therapy and any emerging resistance mutations is also crucial, as it may necessitate a change in therapeutic strategy. These therapies are preferred due to their targeted mechanism of action, which tends to result in higher response rates and more favorable side effect profiles compared to traditional chemotherapy.
NSCL-32	For an advanced lung adenocarcinoma patient with no prior treatment and BRAF V600E fusion mutations, targeted therapy is the preferred treatment option. The recommended therapies are as follows: Dabrafenib (Tafinlar) plus Trametinib (Mekinist): This combination targets the BRAF V600E mutation and has shown significant efficacy in treating lung adenocarcinoma patients with this specific mutation. Dabrafenib is a BRAF inhibitor, and Trametinib is a MEK inhibitor. Together, they block the signaling pathways that contribute to cancer cell growth and survival. Monotherapy with Dabrafenib: In some cases, especially if the combination therapy is not tolerated, monotherapy with Dabrafenib can be considered. These targeted therapies are preferred because they specifically inhibit the aberrant signaling caused by the BRAF V600E mutation, leading to more effective and tailored treatment outcomes for patients with this genetic alteration.
NSCL-33	For a patient with advanced lung adenocarcinoma and an ETV6-NTRK3 gene fusion, the preferred therapy would involve targeting the NTRK (neurotrophic tyrosine receptor kinase) fusion. The main options for this specific genetic alteration are: Larotrectinib (Vitrakvi): This is a highly selective TRK inhibitor designed to treat solid tumors with NTRK gene fusions. It is often the first choice due to its specificity and efficacy in targeting NTRK fusions. Entrectinib (Rozlytrek): Another TRK inhibitor that targets NTRK gene fusions as well as ROS1 and ALK gene fusions. Entrectinib is effective in treating tumors with these genetic alterations, including those in the central nervous system. Both of these therapies have shown substantial efficacy in patients with NTRK fusion-positive cancers, including lung adenocarcinoma. The choice between the two may depend on specific patient factors, such as the presence of brain metastases (where entrectinib might be preferred due to its better CNS penetration) and the side effect profile. It's essential to have a multidisciplinary team approach, involving oncologists, genetic counselors, and other specialists, to tailor the treatment plan to the patient's specific clinical scenario. Regular monitoring and adjustments based on response and tolerance to the therapy are also crucial.
NSCL-34	For a patient with advanced lung adenocarcinoma harboring a MET c.3028 + 2T>C mutation, targeted therapy options are particularly relevant. The MET exon 14 skipping mutations are known to be sensitive to MET inhibitors. Here are the preferred therapies: Capmatinib (Tabrecta): Capmatinib is a MET inhibitor specifically approved for the treatment of metastatic non-small cell lung cancer (NSCLC) with MET exon 14 skipping mutations. Clinical trials have shown significant efficacy in patients with this mutation. Tepotinib (Tepmetko): Tepotinib is another MET inhibitor approved for the treatment of adult patients with metastatic NSCLC harboring MET exon 14 skipping alterations. It has demonstrated efficacy in clinical studies. Crizotinib (Xalkori): Crizotinib, initially approved for ALK-positive NSCLC, has also shown activity against MET exon 14 skipping mutations. It can be considered when other MET inhibitors are not available or suitable. These therapies specifically target the aberrant MET signaling pathway caused by the exon 14 skipping mutation, which is believed to drive cancer growth and progression in this subset of lung adenocarcinoma patients. Additional Considerations: Biopsy and Molecular Testing: It is crucial to confirm the presence of the MET exon 14 skipping mutation through comprehensive genomic profiling. This ensures the mutation is accurately identified and targeted therapy is appropriate. Combination Therapy: In certain cases, combining MET inhibitors with other treatments such as immune checkpoint inhibitors or chemotherapy may be considered, depending on the patient’s overall condition and response to initial treatments. Monitoring and Management of Side Effects: MET inhibitors can have side effects, including gastrointestinal issues, edema, and liver function abnormalities. Regular monitoring and supportive care are essential to manage these effects. Emerging Therapies and Clinical Trials: Patients may also consider enrolling in clinical trials investigating new MET inhibitors or combination therapies that might provide additional benefit. Consulting with a medical oncologist who specializes in lung cancer and is aware of the latest research and clinical trials is crucial for optimal treatment planning.
NSCL-35	For a patient with advanced lung adenocarcinoma harboring a CCDC6::RET gene fusion mutation, the preferred therapies would typically include targeted therapies specifically designed to inhibit RET (rearranged during transfection) kinase activity. The main options include: Selpercatinib (Retevmo): This is a highly selective RET inhibitor that has shown efficacy in patients with RET fusion-positive non-small cell lung cancer (NSCLC). It has been approved by the FDA for this indication. Pralsetinib (Gavreto): Another selective RET inhibitor, also approved by the FDA for the treatment of RET fusion-positive NSCLC. These therapies are preferred due to their targeted action against RET gene fusions, leading to more effective management of the cancer with potentially fewer side effects compared to traditional chemotherapy. In addition to targeted therapies, treatment plans might also consider: Clinical trials: Participation in clinical trials exploring new RET inhibitors or combination therapies might be an option, depending on the patient's eligibility and availability of trials. It is important for the treating oncologist to evaluate the patient’s overall health, prior treatment history, and specific characteristics of the tumor before deciding on the best course of therapy. Regular monitoring and adjustments may also be necessary based on the patient's response and any side effects experienced.
NSCL-36	For a patient with advanced lung adenocarcinoma harboring ERBB2 (HER2) A775_G776insYVMA mutations who has shown recurrence after treatment with cisplatin and pemetrexed, the following targeted therapies are preferred: 1. Trastuzumab Deruxtecan (T-DXd): This is an antibody-drug conjugate specifically targeting HER2. It has shown significant efficacy in patients with HER2-mutant non-small cell lung cancer (NSCLC). 2. Afatinib: This is a pan-HER tyrosine kinase inhibitor (TKI) that has shown activity against HER2 mutations. While it is not as specific as trastuzumab deruxtecan, it can still be effective in this setting. 3. Poziotinib: Another TKI that has shown promising results in clinical trials for HER2-mutant NSCLC, particularly for those with exon 20 insertion mutations. 4. Mobocertinib (TAK-788): This is another TKI that has been shown to be effective against HER2 exon 20 insertion mutations. 5. Neratinib: A pan-HER inhibitor that has shown activity in HER2-mutant lung cancer in clinical trials. 6. Clinical Trials: Consideration should also be given to enrolling the patient in clinical trials investigating novel agents targeting HER2 or other pathways relevant to their specific mutation profile. It is essential to have a multidisciplinary team involved in the decision-making process, including oncologists with expertise in targeted therapies and genetic counselors, to determine the most appropriate treatment plan based on the patient’s overall health, previous treatments, and specific mutation profile.

Appendix F: Questions and responses: ChatGPT-4+PDF+Prompt

**Table 8:** ChatGPT4+PDF+Prompt Responses

Q#	ChatGPT4+Prompt Response
NSCL-21	For an advanced lung adenocarcinoma patient with EGFR p. L858R mutations, the preferred therapy is: Osimertinib Evidence Supporting the Recommendation: Osimertinib is recommended as the preferred first-line therapy for patients with EGFR mutations, including p. L858R, due to its efficacy in improving progression-free survival and overall survival compared to earlier-generation EGFR inhibitors. Citation: ASCO Guidelines: "Osimertinib is the preferred first-line therapy for patients with EGFR p. L858R mutations"​​. NCCN Guidelines: "Osimertinib is recommended as the preferred treatment for EGFR mutation-positive NSCLC patients"​​.
NCSL-22	For an advanced lung adenocarcinoma patient with EGFR p.L858R mutations who has demonstrated progression on osimertinib and has multiple metastases, the preferred therapies according to the latest guidelines are: Amivantamab-vmjw + Carboplatin + Pemetrexed Evidence Supporting the Recommendation: The combination of amivantamab-vmjw, a bispecific antibody targeting EGFR and MET, with carboplatin and pemetrexed has shown to improve progression-free survival (PFS) compared to chemotherapy alone. This is based on the MARIPOSA-2 phase 3 randomized trial, which demonstrated a longer median PFS for the combination therapy group compared to the chemotherapy group. Citation: NCCN NCSL 2024 Guidelines​​. Local Therapy (if limited progression) Evidence Supporting the Recommendation: For patients with asymptomatic progression or symptomatic systemic progression limited in nature (oligoprogression), definitive local therapy such as stereotactic ablative radiotherapy (SABR) or surgery is recommended. This approach is also supported by the NCCN guidelines for specific scenarios including CNS progression. Citation: NCCN NCSL 2024 Guidelines​​. Re-biopsy and Testing for Resistance Mechanisms Evidence Supporting the Recommendation: If systemic progression occurs, a biopsy is recommended to rule out transformation to small cell lung cancer (SCLC) and to evaluate mechanisms of resistance, which may guide subsequent therapy choices. Citation: NCCN NCSL 2024 Guidelines​​. These options align with the latest NCCN and ASCO guidelines for managing advanced NSCLC with EGFR mutations post-osimertinib progression​​​​.
NCSL-23	For an advanced lung adenocarcinoma patient with progression on afatinib and the presence of EGFR p.L858R and T790M mutations, the preferred therapy according to the latest guidelines is: Osimertinib Evidence Supporting the Recommendation: Osimertinib is specifically recommended for patients with EGFR T790M mutations who have progressed on first- or second-generation EGFR inhibitors such as afatinib. It has shown efficacy in targeting both the L858R and T790M mutations, providing a significant clinical benefit. Citation: NCCN Guidelines: "For patients with EGFR T790M mutation-positive NSCLC who have progressed on prior EGFR TKI therapy, osimertinib is the preferred treatment option." ​​ ASCO Guidelines: "Osimertinib is the preferred therapy for patients with EGFR T790M mutations after progression on previous EGFR inhibitors." ​​
NSCL-24	For an advanced lung adenocarcinoma patient with G719X mutations, the preferred therapies based on the latest guidelines are as follows: Preferred First-Line Therapies Afatinib Evidence Supporting the Recommendation: Afatinib has shown a response rate of 77.8% in patients with G719X mutations . Citation: NCCN NCSL 2024 Guidelines, Page NSCL-24; ASCO With Driver 2024 Guidelines, Page 42. Osimertinib Evidence Supporting the Recommendation: Osimertinib demonstrated a median progression-free survival (PFS) of 8.2 months with an objective response rate of 53% in patients with G719X mutations . Citation: NCCN NCSL 2024 Guidelines, Page NSCL-24; ASCO With Driver 2024 Guidelines, Page 42. Other Recommended Therapies Erlotinib Evidence Supporting the Recommendation: Erlotinib is listed as a recommended option, though it is not the preferred choice . Citation: NCCN NCSL 2024 Guidelines, Page NSCL-24. Gefitinib Evidence Supporting the Recommendation: Gefitinib is included as an alternative therapy . Citation: NCCN NCSL 2024 Guidelines, Page NSCL-24. Dacomitinib Evidence Supporting the Recommendation: Dacomitinib is mentioned as an alternative option . Citation: NCCN NCSL 2024 Guidelines, Page NSCL-24. The preference for afatinib and osimertinib is due to their higher efficacy in patients with uncommon EGFR mutations, including G719X, based on clinical trial data and guideline recommendations .
NSCL-25	For a patient with advanced lung adenocarcinoma harboring EGFR Exon 20 Asp770_Asn771ins mutations, the preferred therapy is: Amivantamab Evidence Supporting the Recommendation: Amivantamab has shown efficacy in patients with EGFR Exon 20 insertions, including Asp770_Asn771ins mutations. Clinical trials have demonstrated significant antitumor activity in this subgroup of patients, providing a viable treatment option. Citation: NCCN Guidelines, page 130​​. ASCO Guidelines, page 36​​.
NSCL-27	For a patient with advanced lung adenocarcinoma with EML4-ALK fusion mutations, the preferred therapies based on the latest NCCN and ASCO guidelines are: Alectinib Evidence Supporting the Recommendation: Alectinib has demonstrated superior efficacy compared to crizotinib in the first-line setting for ALK-positive NSCLC, with improved progression-free survival (PFS) and a better safety profile. It is recommended as a preferred first-line therapy for ALK rearrangement-positive metastatic NSCLC. Citation: NCCN NCSL 2024 Guidelines , ASCO With Driver 2024 Guidelines . Brigatinib Evidence Supporting the Recommendation: Brigatinib has shown superior PFS compared to crizotinib in clinical trials for ALK-positive NSCLC. It is also recommended as a preferred first-line therapy. Citation: NCCN NCSL 2024 Guidelines , ASCO With Driver 2024 Guidelines . Lorlatinib Evidence Supporting the Recommendation: Lorlatinib has demonstrated significant intracranial efficacy and systemic responses in patients with advanced ALK-positive NSCLC, including those who have progressed on first- and second-generation ALK inhibitors. It is recommended as a preferred first-line therapy. Citation: NCCN NCSL 2024 Guidelines , ASCO With Driver 2024 Guidelines . These therapies are categorized as first-line treatments for patients with ALK rearrangements, with alectinib, brigatinib, and lorlatinib being the preferred options due to their efficacy and safety profiles.
NSCL-30	For a patient with advanced lung adenocarcinoma with a CD74-ROS1 fusion mutation and no prior treatment, the preferred therapies according to the latest guidelines are: Crizotinib Evidence Supporting the Recommendation: Crizotinib has demonstrated significant efficacy in ROS1-positive NSCLC patients. In multiple studies, it has shown an objective response rate (ORR) of approximately 70-80%, with median progression-free survival (PFS) ranging from 15.9 to 20 months and median overall survival (OS) of 51.4 months. Citation: NCCN NCSL 2024 Guidelines​​ . Entrectinib Evidence Supporting the Recommendation: Entrectinib has shown a high ORR of 77% in ROS1-positive NSCLC patients, with a median PFS of 15.7 months. It is particularly noted for its effectiveness in patients with CNS metastases. Citation: NCCN NCSL 2024 Guidelines​​ . Repotrectinib Evidence Supporting the Recommendation: Repotrectinib is a next-generation ROS1 inhibitor that has shown a confirmed ORR of 79% in ROS1 TKI-naïve patients and 38% in those previously treated with a ROS1 TKI. The median PFS was 35.7 months in TKI-naïve patients. Citation: NCCN NCSL 2024 Guidelines​​ . These therapies are recommended based on their efficacy and the clinical trial data supporting their use in ROS1-positive NSCLC patients.
NSCL-32	For an advanced lung adenocarcinoma patient with no prior treatment and BRAF V600E mutations, the preferred therapies are: Dabrafenib plus Trametinib Evidence Supporting the Recommendation: This combination is approved by the FDA for the treatment of metastatic NSCLC with BRAF V600E mutations. Clinical data from a phase 2 trial showed an overall response rate of 63.9%, with a median progression-free survival (PFS) of 10.8 months and a median overall survival (OS) of 17.3 months in treatment-naïve patients​​. Citation: NCCN NCSL 2024 Guidelines, MS-87, NSCL-32. Encorafenib plus Binimetinib Evidence Supporting the Recommendation: This combination is also FDA-approved for metastatic NSCLC with BRAF V600E mutations. A phase 2 study demonstrated an objective response rate (ORR) of 75% in treatment-naïve patients with a median PFS that was not estimable at the time of the study and a favorable safety profile​​​​. Citation: NCCN NCSL 2024 Guidelines, MS-87, NSCL-32. Both combinations are supported by clinical evidence and are included as preferred first-line therapy options in the NCCN guidelines for patients with BRAF V600E mutation-positive advanced or metastatic NSCLC​​.
NSCL-33	For an advanced lung adenocarcinoma patient with ETV6-NTRK3 mutations, the preferred therapy would be: Larotrectinib Evidence Supporting the Recommendation: Larotrectinib is recommended for patients with NTRK gene fusions, including ETV6-NTRK3, due to its demonstrated efficacy in targeting these mutations. Citation: NCCN Guidelines for Non-Small Cell Lung Cancer, 2024​​; ASCO Guidelines for NSCLC with Driver Mutations, 2024​​. Additionally: Entrectinib Evidence Supporting the Recommendation: Entrectinib is also an effective treatment option for patients with NTRK gene fusions, providing another targeted therapy option. Citation: NCCN Guidelines for Non-Small Cell Lung Cancer, 2024​​; ASCO Guidelines for NSCLC with Driver Mutations, 2024​​.
NSCL-34	For an advanced lung adenocarcinoma patient with MET c.3028 + 2T>C mutations, which result in MET exon 14 skipping, the following therapies are preferred: Capmatinib Evidence Supporting the Recommendation: Capmatinib is an oral MET tyrosine kinase inhibitor (TKI) specifically indicated for patients with MET exon 14 skipping mutations. Clinical data from the GEOMETRY study demonstrated an overall response rate (ORR) of 68% and a median progression-free survival (PFS) of 12.4 months in treatment-naive patients​​. Citation: NCCN NCSL 2024 Guidelines Tepotinib Evidence Supporting the Recommendation: Tepotinib is another MET TKI approved for treating patients with MET exon 14 skipping mutations. The VISION study showed an ORR of 46% and a median PFS of 8.5 months in patients with stage IV NSCLC​​. Citation: NCCN NCSL 2024 Guidelines Both drugs are recommended as first-line monotherapy options for MET exon 14 skipping mutation-positive metastatic NSCLC​​​​. Preferred Therapy Structure: Drug Name: Capmatinib Evidence Supporting the Recommendation: High efficacy in MET exon 14 skipping mutation-positive patients with a significant ORR and PFS. Citation: NCCN NCSL 2024 Guidelines, ASCO With Driver 2024 Guidelines Drug Name: Tepotinib Evidence Supporting the Recommendation: Demonstrated efficacy with substantial ORR and PFS in clinical trials for MET exon 14 skipping mutation-positive patients. Citation: NCCN NCSL 2024 Guidelines, ASCO With Driver 2024 Guidelines
NSCL-35	For an advanced lung adenocarcinoma patient with CCDC6::RET gene fusion mutations, the preferred therapies are: Selpercatinib Evidence Supporting the Recommendation: Selpercatinib is a highly selective RET kinase inhibitor showing substantial efficacy in RET fusion-positive non-small cell lung cancer (NSCLC). Clinical trials have demonstrated significant response rates and progression-free survival in patients treated with selpercatinib. Citation: NCCN NCSL 2024 Guidelines, ASCO With Driver 2024 Guidelines​​​​ Pralsetinib Evidence Supporting the Recommendation: Pralsetinib is another selective RET inhibitor recommended for patients with RET fusion-positive NSCLC. It has shown efficacy in improving clinical outcomes with manageable toxicity profiles in clinical studies. Citation: NCCN NCSL 2024 Guidelines, ASCO With Driver 2024 Guidelines​​​​ These therapies align with the latest guidelines and are considered first-line treatments for patients with RET fusion-positive NSCLC.
NSCL-36	For a patient with advanced lung adenocarcinoma harboring ERBB2 A775_G776insYVMA mutations who has demonstrated recurrence after treatment with cisplatin and pemetrexed, the following therapy is recommended: Drug Name: Trastuzumab deruxtecan Evidence Supporting the Recommendation: Trastuzumab deruxtecan is recommended for patients with ERBB2 (HER2) mutations, particularly in cases where the cancer has progressed after platinum-based chemotherapy. This recommendation is based on its efficacy demonstrated in clinical trials showing significant tumor response and disease control rates in this subset of NSCLC patients. Citation: ASCO Guidelines: "Trastuzumab deruxtecan is recommended for patients with ERBB2 (HER2) mutations after the failure of platinum-based chemotherapy"​​. NCCN Guidelines: "For patients with advanced NSCLC with ERBB2 mutations who have progressed after initial chemotherapy, trastuzumab deruxtecan is a preferred option"​​.

Appendix G: Questions and responses: GPT-4+Prompt

**Table 9:** GPT4+Prompt Responses

Q#	ChatGPT4+Prompt Response
NSCL-21	For a patient with advanced lung adenocarcinoma harboring an EGFR p.L858R mutation, the preferred therapy would be: Osimertinib Evidence Supporting the Recommendation: Osimertinib, a third-generation EGFR-TKI, has demonstrated superior efficacy in terms of progression-free survival and overall survival compared to first-generation EGFR-TKIs. It is particularly effective against both common sensitizing mutations (exon 19 deletions and L858R) and T790M resistance mutations. Citation: NCCN Clinical Practice Guidelines in Oncology: Non–Small Cell Lung Cancer, Version 6.2024 Therapy for Stage IV Non–Small Cell Lung Cancer With Driver Alterations: ASCO Living Guideline, Version 2023.3
NCSL-22	For a patient with advanced lung adenocarcinoma harboring an EGFR p.L858R mutation, who has shown progression on osimertinib and has multiple metastases, the preferred therapy would be: Amivantamab and Lazertinib Combination Evidence Supporting the Recommendation: For patients with EGFR-mutant NSCLC who have progressed on osimertinib, amivantamab (an EGFR-MET bispecific antibody) combined with lazertinib (a third-generation EGFR inhibitor) has shown promising efficacy. This combination targets multiple resistance mechanisms that can arise after osimertinib treatment, offering a potential therapeutic option for patients with acquired resistance. Citation: Non–Small Cell Lung Cancer, Version 6.2024, NCCN Clinical Practice Guidelines in Oncology. Alternatively, if amivantamab and lazertinib are not available or suitable: Chemotherapy (Platinum-based doublet with or without bevacizumab) Evidence Supporting the Recommendation: In cases where targeted therapies are no longer effective, platinum-based chemotherapy (such as carboplatin or cisplatin combined with pemetrexed) remains a standard approach. Bevacizumab may be added for patients with non-squamous histology, as it has been shown to improve outcomes in combination with chemotherapy. Citation: Non–Small Cell Lung Cancer, Version 6.2024, NCCN Clinical Practice Guidelines in Oncology. These recommendations align with current clinical guidelines and consider the progression on first-line osimertinib and the need for subsequent lines of therapy.
NCSL-23	For an advanced lung adenocarcinoma patient with progression on afatinib and harboring EGFR p.L858R and T790M mutations, the preferred therapy is osimertinib. Drug Name: Osimertinib Evidence Supporting the Recommendation: Osimertinib is a third-generation EGFR tyrosine kinase inhibitor (TKI) specifically effective against EGFR T790M mutations, which are a common mechanism of resistance to first- and second-generation EGFR TKIs such as afatinib. Clinical trials, including the AURA3 study, have demonstrated that osimertinib provides significant improvements in progression-free survival and overall response rates in patients with T790M-positive NSCLC. Citation: NCCN Clinical Practice Guidelines in Oncology, Non-Small Cell Lung Cancer, Version 6.2024; ASCO Living Guideline, Version 2023.3
NSCL-24	For an advanced lung adenocarcinoma patient with G719X mutations, the preferred therapy options are based on the latest guidelines from NCCN and ASCO. These guidelines emphasize the use of targeted therapies for patients with specific genetic alterations. Preferred Therapy: Osimertinib Evidence Supporting the Recommendation: Osimertinib is an EGFR tyrosine kinase inhibitor (TKI) that has shown efficacy in patients with uncommon EGFR mutations, including G719X. It is preferred due to its efficacy and favorable safety profile. Citation: NCCN Clinical Practice Guidelines in Oncology: Non–Small Cell Lung Cancer, Version 6.2024. Afatinib Evidence Supporting the Recommendation: Afatinib has demonstrated activity against uncommon EGFR mutations such as G719X. It is recommended based on its proven effectiveness in this subset of patients. Citation: ASCO Living Guideline, Version 2023.3. These recommendations align with the current guidelines, which prioritize targeted therapies for patients with driver mutations in NSCLC.
NSCL-25	For an advanced lung adenocarcinoma patient with EGFR Exon 20 Asp770_Asn771ins mutations, the preferred therapy would be: Amivantamab-vmjw Evidence Supporting the Recommendation: Amivantamab is a fully human EGFR-MET bispecific antibody indicated for the treatment of patients with NSCLC harboring EGFR Exon 20 insertion mutations. Clinical trials have demonstrated its efficacy and safety in this patient population, making it a recommended option. Citation: NCCN Clinical Practice Guidelines in Oncology, Non–Small Cell Lung Cancer, Version 6.2024 Additionally, another recommended therapy option is: Mobocertinib Evidence Supporting the Recommendation: Mobocertinib is an oral tyrosine kinase inhibitor specifically designed to target EGFR Exon 20 insertion mutations. Clinical studies have shown that it provides significant clinical benefit in patients with these mutations. Citation: NCCN Clinical Practice Guidelines in Oncology, Non–Small Cell Lung Cancer, Version 6.2024 These therapies are aligned with the latest NCCN guidelines and are considered standard care for patients with this specific genetic alteration.
NSCL-27	For an advanced lung adenocarcinoma patient with EML4-ALK fusion mutations, the preferred therapies according to the latest guidelines are: Alectinib Evidence Supporting the Recommendation: Alectinib has demonstrated superior efficacy in terms of progression-free survival and overall survival compared to crizotinib, and it has a more favorable side effect profile. Citation: NCCN Guidelines, Version 6.2024; ASCO Living Guideline, Version 2023.3 Brigatinib Evidence Supporting the Recommendation: Brigatinib has shown significant improvements in progression-free survival over crizotinib and is effective in patients with central nervous system metastases. Citation: NCCN Guidelines, Version 6.2024; ASCO Living Guideline, Version 2023.3 Lorlatinib Evidence Supporting the Recommendation: Lorlatinib is recommended particularly for patients who have progressed on prior ALK inhibitors, with demonstrated activity against brain metastases and a favorable response rate. Citation: NCCN Guidelines, Version 6.2024; ASCO Living Guideline, Version 2023.3 These therapies are aligned with the NCCN and ASCO guidelines for the treatment of NSCLC with ALK rearrangements and are considered the most effective options for this specific patient population.
NSCL-30	For an advanced lung adenocarcinoma patient with no prior treatment and CD74-ROS1 fusion mutations, the preferred therapies are: Entrectinib Crizotinib Entrectinib Evidence Supporting the Recommendation: Entrectinib is an ROS1 inhibitor with demonstrated efficacy in ROS1-positive NSCLC, showing high response rates and durability of response. Citation: NCCN Guidelines, Version 6.2024 Crizotinib Evidence Supporting the Recommendation: Crizotinib, another ROS1 inhibitor, has been shown to be effective in ROS1-rearranged NSCLC, with substantial clinical benefit and prolonged progression-free survival. Citation: NCCN Guidelines, Version 6.2024 These recommendations are based on the latest NCCN guidelines, which highlight both entrectinib and crizotinib as preferred options for patients with ROS1 rearrangements.
NSCL-32	For a patient with advanced lung adenocarcinoma harboring BRAF V600E mutations, the preferred therapy aligns with the latest NCCN and ASCO guidelines. Dabrafenib + Trametinib Evidence Supporting the Recommendation: The combination of Dabrafenib, a BRAF inhibitor, and Trametinib, a MEK inhibitor, has shown significant efficacy in patients with BRAF V600E mutations. Clinical trials have demonstrated improved response rates and progression-free survival compared to historical controls. Citation: Non–Small Cell Lung Cancer, Version 6.2024, NCCN Clinical Practice Guidelines in Oncology; Therapy for Stage IV Non–Small Cell Lung Cancer With Driver Alterations: ASCO Living Guideline, Version 2023.3
NSCL-33	For an advanced lung adenocarcinoma patient with ETV6-NTRK3 mutations, the preferred therapies are: Larotrectinib Evidence Supporting the Recommendation: Larotrectinib is a highly selective TRK inhibitor shown to be effective in patients with NTRK gene fusions, including ETV6-NTRK3, leading to significant and durable responses in advanced solid tumors. Citation: Non–Small Cell Lung Cancer, Version 6.2024, NCCN Clinical Practice Guidelines in Oncology Entrectinib Evidence Supporting the Recommendation: Entrectinib is another TRK inhibitor that targets NTRK gene fusions. It has demonstrated efficacy and safety in treating patients with advanced solid tumors harboring NTRK fusions, including ETV6-NTRK3. Citation: Non–Small Cell Lung Cancer, Version 6.2024, NCCN Clinical Practice Guidelines in Oncology
NSCL-34	For a patient with advanced lung adenocarcinoma harboring MET exon 14 skipping mutation (e.g., MET c.3028 + 2T>C), the following targeted therapies are recommended: Capmatinib Evidence Supporting the Recommendation: Capmatinib is an FDA-approved MET inhibitor specifically for patients with metastatic NSCLC harboring MET exon 14 skipping mutations. Clinical trials have shown significant efficacy in terms of response rates and progression-free survival. Citation: NCCN Clinical Practice Guidelines in Oncology: Non–Small Cell Lung Cancer, Version 6.2024. Tepotinib Evidence Supporting the Recommendation: Tepotinib has demonstrated clinical benefit in patients with MET exon 14 skipping mutations, with studies indicating robust and durable responses. It is also FDA-approved for this specific genetic alteration. Citation: NCCN Clinical Practice Guidelines in Oncology: Non–Small Cell Lung Cancer, Version 6.2024. Both therapies are aligned with current guidelines and represent preferred treatment options for patients with MET exon 14 skipping mutations.
NSCL-35	For a patient with advanced lung adenocarcinoma harboring CCDC6::RET gene fusion mutations, the preferred therapies are as follows: Selpercatinib Evidence Supporting the Recommendation: Selpercatinib is a selective RET kinase inhibitor that has shown significant efficacy in patients with RET fusion-positive non-small cell lung cancer (NSCLC). Clinical trials have demonstrated high response rates and durable responses with a favorable safety profile. Citation: NCCN Guidelines Version 6.2024, ASCO Living Guideline Version 2023.3 Pralsetinib Evidence Supporting the Recommendation: Pralsetinib is another selective RET inhibitor that has been proven effective for patients with RET fusion-positive NSCLC. It has shown robust clinical activity and a manageable safety profile in clinical studies. Citation: NCCN Guidelines Version 6.2024, ASCO Living Guideline Version 2023.3 These therapies align with the latest NCCN and ASCO guidelines and are recommended as first-line treatments for patients with RET fusion-positive NSCLC.
NSCL-36	For a patient with advanced lung adenocarcinoma harboring an ERBB2 (HER2) A775_G776insYVMA mutation who has shown recurrence after treatment with cisplatin and pemetrexed, the following therapy is recommended: Trastuzumab deruxtecan (Enhertu) Evidence Supporting the Recommendation: Trastuzumab deruxtecan, an antibody-drug conjugate targeting HER2, has demonstrated significant efficacy in patients with ERBB2 mutations in non-small cell lung cancer. Clinical trials have shown high response rates and durable responses in this patient population. Citation: NCCN Guidelines Version 6.2024 for Non-Small Cell Lung Cancer; ASCO Living Guideline, Version 2023.3 for Therapy for Stage IV Non-Small Cell Lung Cancer With Driver Alterations. This recommendation is based on the specific genetic alteration present in the patient's tumor and aligns with current clinical practice guidelines for targeted therapy in NSCLC with ERBB2 mutations.
